# Molecular Design and Nanoarchitectonics of Inorganic–Organic Hybrid Sol–Gel Systems for Antifouling Coatings

**DOI:** 10.3390/gels10120768

**Published:** 2024-11-25

**Authors:** Markus Bös, Ludwig Gabler, Willi Max Leopold, Max Steudel, Mareike Weigel, Konstantin Kraushaar

**Affiliations:** 1 Institut für Anorganische Chemie, TU Bergakademie Freiberg, 09596 Freiberg, Germany; markus.boes@chemie.tu-freiberg.de (M.B.); ludwig.gabler@chemie.tu-freiberg.de (L.G.); willi-max.leopold@chemie.tu-freiberg.de (W.M.L.); max.steudel@student.tu-freiberg.de (M.S.); mareike.weigel@chemie.tu-freiberg.de (M.W.); 2 Zentrum für Effiziente Hochtemperatur-Stoffwandlung (ZeHS), TU Bergakademie Freiberg, 09596 Freiberg, Germany

**Keywords:** antifouling, sol–gel, xerogel, inorganic–organic hybrid coating, fouling resist, fouling release, (biocidal) additives, surface catalysis

## Abstract

Environmental protection, especially fouling protection, is a very topical and wide-ranging issue. This review explores the development, molecular design, and nanoarchitectonics of sol–gel-based hybrid coatings for antifouling applications. These coatings combine inorganic and organic materials, offering enhanced stability and adaptability, making them ideal for protecting surfaces from fouling. This review covers key antifouling strategies from the past decade, including biocidal additives, fouling resistance, release mechanisms, and surface topological modifications. The sol–gel hybrid systems prevent biofilm formation and organism attachment by leveraging molecular interactions, making them particularly useful in marine environments. Additionally, the study emphasizes the coatings’ environmental benefits, as they offer a potential alternative to traditional toxic antifouling methods. Overall, this research underscores the importance of sol–gel technologies in advancing eco-friendly antifouling solutions.

## 1. Introduction

**Figure 1:**
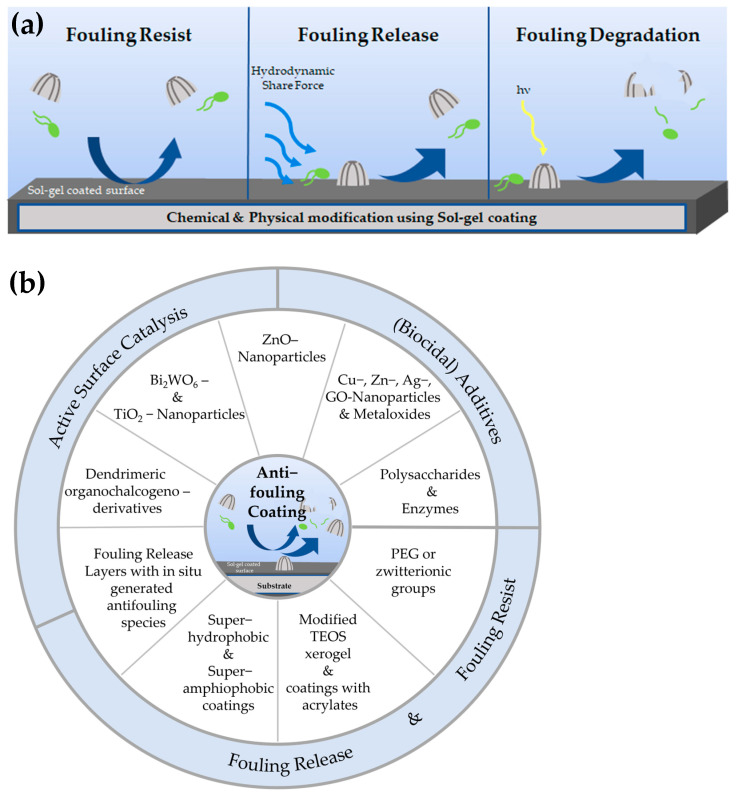
(**a**) Graphical representation of possible antifouling methods with sol-gel coated surfaces, (**b**) Graphical outline of the key components in sol–gel chemistry for antifouling coatings.

Sol–gel coatings have attracted considerable interest because of their versatility and chemical durability, making them appropriate for a wide range of applications, including antifouling coatings. Marine fouling is a widespread problem characterized by the fast attachment of organic compounds to the submerged surfaces of ships. In a matter of minutes, ions, polysaccharides, proteins, glycoproteins, and humic and fulvic acids in the water adhere to these surfaces by adsorption [1,2]. Due to the formation of electrostatic forces and van der Waals interactions, microorganisms begin to accumulate after just a few hours. Through their excretion of extracellular polymeric substances, a biofilm is formed [2]. Biofilms, normally consisting of extracellular polymeric compounds, function as a living environment for diatom spores and other microorganisms. These microorganisms can undergo development within a matter of days [1]. Biofilms provide several benefits for larger species like barnacles, mussels, and macroalgae, allowing them to colonize surfaces in as little as one month. The benefits are protection against environmental contaminants, facilitation of nutrient exchange, and enhanced resilience to biocides. Also, the biofilm modifies the surface chemistry nearby, increasing its attractivity for the attachment of larger organisms. Effective antifouling solutions are essential because of the quick and intricate growth of fouling [2]. The molecular design and nanoarchitectonics of sol–gel coatings show great potential because of their hybrid inorganic–organic composition. Nanoarchitectonics describes the universal approach of creating functional materials from the building blocks of atoms and molecules. This review describes topics such as molecular, inorganic, biomolecular, and supramolecular nanoarchitecture to modify such materials at atomic, molecular, and nanoscale levels. These provide very good stability and adaptability. This review examines different antifouling tactics that have been developed in the last ten years. It specifically looks at mechanisms such as the use of biocidal chemicals, fouling release, fouling resistance, and adjustments to surface topology. The review is summarized in a graphical outline shown in Figure 1. The review intends to emphasize the significance and promise of sol–gel coatings in tackling environmental fouling.

Most antifouling approaches can be allocated to one or more of the following mechanisms of action: deployment of (biocidal) additives, fouling release, fouling resist, and active surface catalysis.

## 2. Deployment of (Biocidal) Additives

A broad variety of additives is used to increase the antifouling capabilities of materials produced by sol–gel processes. The use of additives remains a popular method for antifouling in many applications due to its effectiveness, ease of application, and cost-efficiency compared with other alternatives. However, for the additive to be effective, it must be present at the interface between the material and the surrounding medium. The method of achieving this varies depending on the type of polymer matrix used. The additive can be either dissolved, present as a particle, or embedded in the polymer within the coating. To reach the surface, if it is not already present at the interface, it can either slowly migrate or be exposed at the interface by removal of material. Both mechanisms can lead to the leaching of the additive and polymer into the surrounding medium and accumulation in it. The goal of the addition is to improve the antifouling ability of the material. Using stronger biocides will lead to better performance.

Jaiswal et al. synthesized a xerogel by hydrolyzing methyltriethoxysilane (MTES) in the presence of a metal (Ag, Cu, Zn) nitrate salt. Those nanoparticles are well known for their antibacterial activities. Different analyses were conducted, for example, thermogravimetric analysis (TGA) to characterize the curing conditions, as well as nuclear magnetic resonance spectroscopy (NMR), X-ray photoelectron spectroscopy (XPS), and inductively coupled plasma atomic emission spectroscopy (ICP-AES). Contact angle measurements and antibacterial assays describe the antibacterial properties. The main result of the research is that Ag^+^, compared with Zn^2+^ and Cu^2+^, showed the highest reduction in bacterial adhesion within a sol–gel matrix [3]. In another study about the antibacterial assessment of a SiO_2_-hydroxypropylmethyl (HPMC) cellulose hybrid material, silver nanoparticles were tested within a hybrid tetraethoxysilane (TEOS) coating. Angelova et al. carried out two bacterial growth measurements (*B. subtilis* and *E. coli* K12), one by measuring the optical density in the presence of the SiO_2_/HPMC/Ag network and an agar diffusion test. Scanning electron microscopy (SEM) analysis confirmed the presence of 30 nm silver particles on the surface of the coating. With a higher concentration of nanoparticles, an increasing inhibitory effect on the growth of microorganisms was observed [4].

A different approach was chosen by Chapman et al., who synthesized copper nano- and microparticles to inhibit biofouling in its early stages. Those particles were embedded within a methyltrimethoxysilane (MTMS) and butyltrimethoxysilane (BTMS) sol–gel network. Long-term antifouling properties were measured by exposing the materials to a highly fouling marine environment (Galway Bay, Ireland). Results indicate the highest effectiveness for copper nanoparticles to inhibit fouling. A benchmark using commercial antifouling paints highlights the advantages of the tested materials [5].

Not only atomic nanoparticles could be synthesized within or embedded in a xerogel, also the oxides of many metals enhance antifouling properties. In order to stop microfouling, Krupa et al. used environmentally friendly synthesized zinc oxide nanoparticles in combination with a TEOS sol–gel coating. The antifouling effect of the ZnO nanoparticle sol–gel system against the marine biofilm-forming bacteria has been investigated through a standard agar well diffusion technique. The TEOS gel structure was found to be ideal for incorporating nanoparticles. The particles were uniformly distributed and detected by SEM both within and near the surface layers of the coating [6].

Liu et al. synthesized a TEOS/MTMO (3-mercaptopropyltrimethoxysilane) sol and added PDMS (polydimethylsiloxane) to prevent cracking of the final coating. Silver nanoparticles on graphene oxide (GO) were prepared by a thermal reduction method and added to the composite sol. Characterizations like corrosion and antifouling tests were carried out on copper substrates. The authors found a good antibacterial performance toward *E. coli,* and the coatings showed effective antialgae properties against *Phaeodactylum tricornutum*, *Navicula torguatum*, and *Chlorella* [7].

**Figure 2:**
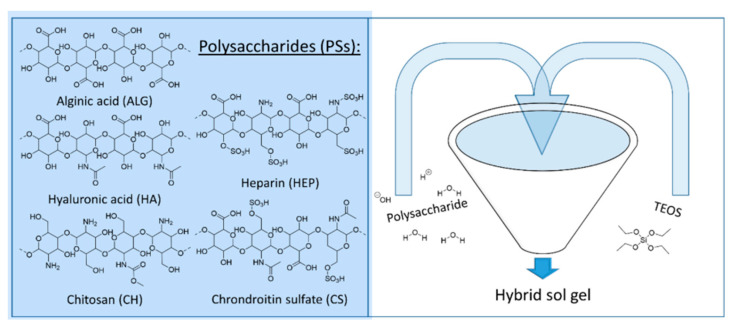
Polysaccharides used by Wanka and Yu et al. to synthesize a hybrid sol–gel network [9].

Besides nanoparticles, different research developments show the incorporation of polysaccharides to be successful in achieving antifouling and fouling release effects. Chitosan, for example, was first incorporated by Dhawade et al. into a sol–gel formulation. Therefore, TEOS and (3-Glycidyloxypropyl)trimethoxysilane (GPTMS) were hydrolyzed in the presence of 0.05 M HCl. In a second step, chitosan, dissolved in acetic acid, was added. Chitosan itself is known for its antimicrobial activity against fungi, algae, and bacteria. Contact angle measurements have been carried out [8]. Wanka et al. developed a hybrid sol–gel coating incorporating naturally occurring polysaccharides. Alginate, chitosan, hyaluronic acid, chondroitin-6-sulfate, and heparin sol–gel coatings have been studied for their antifouling and fouling-release properties. Therefore, each polysaccharide was mixed with water, TEOS, and a catalyst (HCl or NaOH) and hydrolyzed, as shown in Figure 2. As a reference for hydrophobicity octadecyltrichlorosilane monolayer coatings were characterized. The results for the polysaccharide sol–gel coatings show a superior antifouling and fouling release performance compared with the reference. It has been proven that coatings containing polysaccharides rich in anionic groups have better antifouling and fouling-release properties than those with amine groups [9].

**Figure 3:**
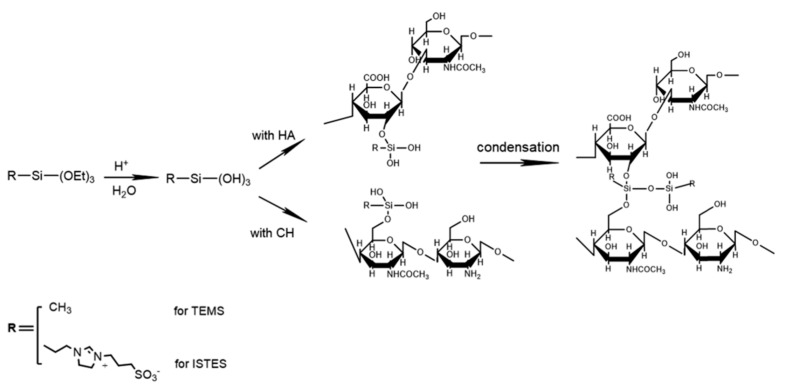
Reaction scheme for the synthesis of LBLHP proposed by Yu et al. [10].

In 2021, the same working group published another paper addressing layer-by-layer-deposited hybrid polymer coatings based on polysaccharides and zwitterionic silanes with marine antifouling properties. Yu et al. incorporated chitosan, alginic acid, hyaluronic acid, and chondroitin sulfate into an acid-catalyzed MTES and 3-(1-(3-(triethoxysilyl)propyl)-4,5-dihydro-1H-imidazol-3-ium-3-yl)propane-1-sulfonate (ISTES) sol–gel mixture. The authors propose layer-by-layer assembling of polysaccharide-based hybrid polymer coatings (LBLHPs) (shown in Figure 3). Final coating solutions were spin-coated on (3-aminopropyl)trimethoxysilane (APTMS) functionalized glass slides or silicon wafers. As a point of reference, self-assembled monolayers on gold substrates were chosen. Resistance of the LBLHPs against nonspecific adsorption of proteins, as well as marine antifouling properties, were tested by surface plasmon resonance measurements (SPR), a dynamic microfluidic attachment assay, and measuring spore settlement densities after 45 min of immersion. It was found that the added polysaccharides, as well as the zwitterionic silane, had a positive effect on antifouling properties. A comparable result to the reference was achieved, making LBLHP a possible contender for novel antifouling coatings [10].

Regina et al. were one of the first to study sol–gel materials in enzyme-based antifouling applications. Therefore, the serine protease *subtilisin* was encapsulated into a sol–gel system and applied to a stainless-steel surface. The sol–gel network was synthesized by mixing Glycerol, GPTES, *n*-propyltriethoxysilane (C3-TES), methylphenyldimethoxysilane (MPDMO), 3-mercaptopropyltriethoxysilane (MTEO) and optionally the enzyme solution. To catalyze the reaction, 0.1 M hydrochloric acid was used. Before application, a small amount of N-Methylaminopropyltrimethoxysilane was added to the mixture because of its suspected activity against *subtilisin* and to increase the porosity of the final coating. Via the colorimetric method, the enzyme activity was determined. Contact angle measurements, as well as bacterial adhesion assays, were carried out. The enzyme activity was retained for nine months, which indicates the potential of immobilized enzymes within a sol–gel coating for antifouling purposes [11]. A combination of the encapsulated endospore-forming strain of *B. licheniformis* and two metal-based phosphate and molybdate corrosion inhibitors were tested by Eduok et al. within an amino-epoxy sol–gel coating. The goal was to observe the inhibition of microbiologically influenced corrosion. Electrochemical impedance spectroscopy (EIS), contact angle measurements, and field tests were used to characterize the TEOS/GPTES/(aminopropylmethylsiloxane)–dimethylsiloxane copolymer coatings anticorrosion and antifouling properties. The endospores increase the surface hydrophobicity and thereby reduce corrosion and fouling. The analysis of the field test results led to an understanding of the fouling release mechanism of the used endospores. The authors have proven that it is possible to incorporate bacterial endospores into the sol–gel network and achieve antifouling properties. [12]

A similar route was researched by Suleiman et al., who studied a TEOS/MTES sol–gel network on mild steel panels. To achieve anticorrosion and antifouling properties, different inorganic inhibitors (Molywhite^®^ 101-ED (MOLY) and Heucophos Zapp^®^ (ZAPP)), as well as *Paenibacillus polymyxa* endospores, were incorporated into the network. Electrochemical impedance measurements, contact angle measurements, and a field test were used to evaluate the antifouling and anticorrosion properties of the coatings. Results indicate that the use of inhibitors and bacteria amplifies the antifouling effect compared with only using bacteria alone. The antifouling mechanism and scalability of the batch is still under investigation [13]. The research of Rathinam et al. highlights that almost any bioactive organic chemical could be introduced within a sol–gel network. Therefore, eugenol was used as a biocide in a TEOS sol–gel system on silicone segments. The potential applications lie within the human body as implants. This plant-based antivirulence agent has the potential to reduce bacterial adhesion and biofilm formation (tested against *P. aeruginosa*). It was proven that eugenol is compatible with sol–gel matrices [14].

An overview of all additives and bioactive materials with antibacterial and antifouling effects is listed in Table 1.

## 3. Fouling Release/Resist

Producing effective biocide-free antifouling solutions is a challenging and costly task. Another approach to prevent fouling is to use fouling resistance systems that employ materials to suppress and/or prevent the adhesion and growth of organisms.

Xerogels offer the possibility to specifically adjust the water wettability and surface energy [15,16,17]. This means that the sol–gel-derived coatings can be used both as fouling-resistant and fouling-release coatings [15,16,18,19]. The three most important parameters for reduced bioadhesion and easy removal of biofouling are surface energy, modulus, and roughness [20,21], whereby the sol–gel technology makes it possible to adjust these three parameters independently of each other in a coating [22]. The contact angle measurement serves as a surface-sensitive method for determining the wetting properties (surface energy), which characterizes the top 4–5 Å of the surface empirically and reproducibly [20,23,24].

**Figure 4:**
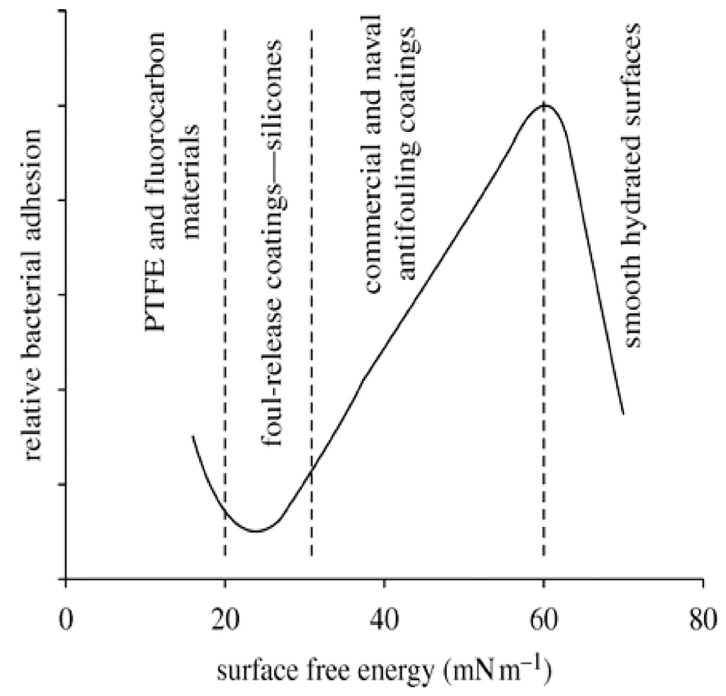
Schematic representation of the Baier curve, in which the relative bacterial adhesion is plotted against the surface energy [22].

Already in 1968, Baier et al. presented the so-called Baier curve, in which the adhesion force of microorganisms is plotted against the surface energy (Figure 4). The bioadhesion is minimal in a range of 20 to 25 mN∙m^−1^ [25,26,27]. Hydrophobic surfaces (fouling release layers) with surface energies close to Baier’s minimum reduce the adhesion of fouling organisms to the surface. The weakly bound organisms can be detached by the action of external shear forces or cleaning, which is due to the low surface energy of the layers and their low modulus of elasticity [22,25,28,29,30,31,32,33]. The share of fouling release coatings on ship hulls was already over 10% in 2015 [33]. A large number of fouling-release coatings have so far been based on silicone elastomers. However, silicones are susceptible to mechanical damage and can grow together with certain types of fouling organisms (e.g., diatoms) [31,34,35].

**Figure 5:**
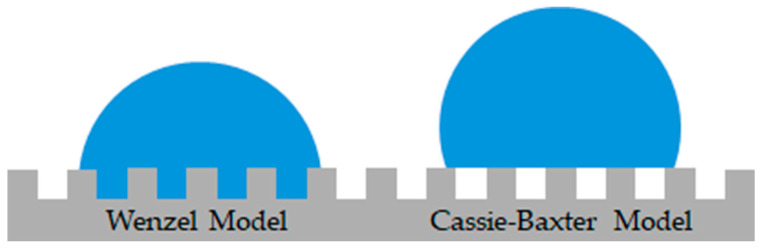
Schematic representation of wetting models (Wenzel left, Cassie–Baxter right).

The surface roughness of a material exerts a significant influence on the wettability of the surface in question. Wenzel proposed that the chemically induced wettability of a surface can be amplified by surface roughness. This effect can be observed in both directions, either increasing the hydrophobicity of chemically hydrophobic surfaces or increasing the hydrophilicity of hydrophilic surfaces [36]. In this simplified model, the liquid will adapt its shape to the surface roughness (Figure 5) [37]. Building on this, Cassie and Baxter examined the wettability of porous and highly rough structures [38]. In these cases, the liquid is suspended on the roughness peaks, with air enclosed in the volumes below the droplet (Figure 5). This reduces the contact area of the liquid with the solid surface, and in some instances, the liquid may be able to roll off the surface [37].

Hydrophobic surfaces can be improved by combining their chemical hydrophobicity with complimentary morphologies. This can be achieved by generating patterns like Pillars [39], Walls [39], and Ridges [21] on the surface and by generating porous structures. One way of creating morphologies is complex etching processes, which are only suitable for some applications. An alternative way is generating an already structured surface. Sol–gel materials can provide nanoporous structures that feature microscale surface features [21].

Fouling-resistance coatings are based on hydrophilic polymers, as these surfaces are resistant to the adsorption of proteins. Protein adhesion is determined by the difference in interfacial energy between the coating surface and water. This difference in interfacial energy is very high for hydrophobic (e.g., polyfluorinated) surfaces and water and lower for hydrophilic surfaces (e.g., with Polyethylene glycol (PEG)) and water. When amphiphilic biomolecules such as proteins come in contact with a hydrophobic surface, a significantly high adhesion to the surface is observed to reduce the interfacial energy. Conversely, the adsorption of biomolecules on hydrophilic surfaces does not lead to any significant thermodynamic advantage as the interfacial energy is already low [40].

However, biofouling also involves other complex processes. Diatom proteins, for example, prefer to bind to surfaces with lower surface energy and can be detached more easily from hydrophilic surfaces [22,41,42]. Different tendencies have been observed in barnacles depending on the species. Older studies have shown that the larvae of *B. amphitrite* prefer to settle on hydrophilic surfaces with high wettability [43,44,45]. The larvae of the barnacle *Balanus improvisus*, on the other hand, which are widely distributed worldwide, prefer to adhere to hydrophobic surfaces [46]. For amphiphilic surfaces with chemical heterogeneity, a reduced colonization of barnacle larvae has also been demonstrated [47].

Several studies on the settlement of zoospores of the green marine alga *Ulva linza* (the most common macrofouling alga on ships) showed a preference for hydrophobic surfaces compared with hydrophilic surfaces [41,42,48,49,50,51]. However, the settled zoospores of *U. linza* adhere more strongly to hydrophilic than to hydrophobic surfaces [51,52,53]. As a result, the spores are generally easier to remove from hydrophobic surfaces [41,42]. Due to these differences in adhesion and fouling release behavior, chemically heterogeneous surfaces were developed, which have both hydrophilic and hydrophobic domains (“chemical mosaic”) [51,54,55].

### 3.1. TEOS Xerogels with Fluorocarbon, Aminopropyl, and Hydrocarbon-Chain-Containing Siloxanes

The organic functionalization of precursors, for example, with alkyl or fluoroalkyl groups, generally results in coatings with a lower surface energy (see Table 2) [22,56]. The system of *n*-octyltriethoxysilane (C8-TES) and TEOS in a molar ratio of 1:1 has already been tested in field trials over several years [22]. This coating is now also known under the trade name AquaFast^®^ [33]. The effect of this coating is based on the low surface energy (21.5 mN m^−1^), which is in the range of Baier’s minimum [33]. This xerogel is characterized by a smooth, chemically homogeneous, crack-free surface, which has a large number of unreacted silanol groups. The coating was applied to a boat in 2006 for testing purposes. After more than three months in water (Irondequoit Bay (NY)), the adhering fouling layer could be easily removed by brushing. The coating was renewed after four seasons (2009) and retained its antifouling and fouling-release properties during this time. Since 2006, the C8-TES/TEOS Xerogel has been applied to over 100 boats in Lake Ontario, with similar performance being achieved. The removal of algae was consistently successful, but the removal of diatoms was dependent on location [22]. The C8-TES/TEOS coating was also tested on the transparent polycarbonate surface of an underwater camera in the Mediterranean (near Pantelleria). The coating resulted in a significant improvement in antifouling properties. Instead of manual cleaning by divers after 40 days, this was only necessary once after three years [19].

**Figure 6:**
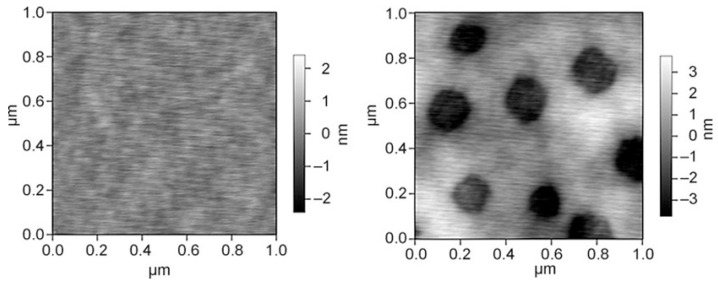
AFM images of 50:50 C8-TES/TEOS coating show a uniform surface (**left**). The AFM images of 1:49:50 C18-TMS/C8-TES/TEOS coating (**right**) show a porous surface with 100–300 nm wide pores that are 3–5 nm deep [33].

The C8-TES/TEOS layer was further modified by the addition of *n*-octadecyltrimethoxysilane (C18-TMS) and is also known as AquaFast Pro^®^ (C18-TMS/C8-TES/TEOS in a 1:49:50 molar ratio) [57]. The modification with *n*-octadecyltrimethoxysilane (C18-TMS) results in chemically and topographically inhomogeneous surfaces with 100–300 nm large pores, whereby the enrichment of C18-TMS at the surface of the pores was shown by means of spatially triggered IR spectroscopy and AFM (Figure 6) [57]. Compared with the original AquaFast^®^ formulation, a greatly improved removal of barnacles (*B. amphitrite*) could be achieved (comparable to conventional silicone coatings PDMSE—poly(dimethylsiloxane) elastomer) [33,57].

**Figure 7:**
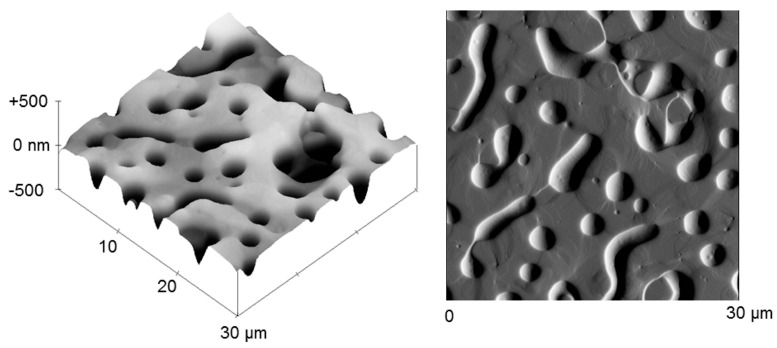
AFM height image of 0.5 mol% PEG in 50:50 TDF/TEOS xerogel (**left**) and AFM image of the 0.5 mol% PEG in 50:50 TDF/TEOS xerogel in amplitude mode (**right**) [22].

Sokolova et al. extended the described system with tridecafluoro-1,1,2,2-tetrahydrooctyltriethoxysilane as a further precursor. C18-TMS, tridecafluorooctyltriethoxysilane (TDF), C8-TES, and TEOS show phase segregation in a range from 1:1:48:50 to 1:9:40:50, forming islands with alkane- and perfluoroalkane-rich regions. The values for the surface energies are within the Baier zone of minimum bioadhesion (21.3–23.1 mN m^−1^) [58]. The coating achieved better results for the fouling release of juvenile barnacles and Ulva sporelings compared with the C18-TMS/C8-TES/TEOS coating [21,58]. The C18-TMS/C8-TES/TEOS and the C18-TMS/TDF/C8-TES/TEOS xerogel were compared with commercial coatings (IS700, IS900 coatings, and T2 silicone standard). The xerogel coating reduced biofouling less well but showed better fouling release performance of microalgae. The good fouling release properties of the two xerogel coatings can be attributed to the formation of a chemical “mosaic”. As already shown by Gunari et al. for the C18-TMS/C8-TES/TEOS layer, the C18-TMS/TDF/C8-TES/TEOS xerogel also has a network of nanoscale pores and regions with higher and lower hydrocarbon content on the surface [57,59]. The C8-TES and the C18-TMS can also be completely replaced by TDF, and PEG can also be introduced into the layer. This results in xerogels with an equimolar ratio of TEOS and TDF with 0.5 mol% PEG, which have features (characteristics, special features) on the order of 10–15 µm. The heterogeneous areas can be detected by AFM (see Figure 7). The fouling release properties of this system were better than those of the C8-TES/TEOS coating [22].

Fouling release coatings based on the concept of topographic heterogeneity were investigated by Destino et al. This special xerogel film based on 3-aminopropyltriethoxysilane (APTES), *n*-octyltriethoxysilane (C8-TES), and TEOS is characterized by chemical, surface-loaded, and topographical heterogeneity on micrometer and nanometer scales. These are caused by hydrophobic-hydrophilic interactions between C8-TES and APTES precursors and/or interactions between the basic APTES and the acidic sites on the Al_2_O_3_ coating substrate. The xerogel film can be divided into three different areas at the 2D and 3D levels. The base layer contains all three precursors and has a thickness of 1.5 µm. Furthermore, there are mesa-shaped structures with a diameter of 2–4 µm, which are enriched with free amine (from APTES) and depleted of the other precursors and rise about 150–400 µm above the base layer. In addition, there are inclusions within the base layer with a diameter of 1–2 μm, which are enriched in hydrogen-bonded amine (from APTES) and depleted of the other species [60].

Another approach to creating textured surfaces is by generating xerogel films on the surface itself. Destino et al. used carboxyethylsilanetriol (COE) to modify TEOS sol–gel formulations. The addition of COE resulted in the formation of complex dendritic features with varying coverage depending on the amount of COE added. Such morphologies are usually generated by photolithography and are therefore not suitable for marine antifouling coatings [60].

Bennet et al. modified the surface properties and fouling characteristics of a series of xerogel coatings. The coatings are based on TEOS and siloxanes with fluorocarbon (3,3,3-trifluropropyltrimethoxysilane—TFP), aminopropyl (APTES, 3-methylaminopropyltrimethoxysilane—MAP, 3-dimethylaminopropyl-trimethoxysilane—DMAP, 3-trimethoxysilyl-propyltrimethyl-ammonium iodide—TMAP), and hydrocarbon groups (phenyltriethoxysilane—PH, C3-TMS, C8-TES, C18-TMS). Coatings with very different wetting properties were produced. The contact angles range from 35° (DMAP/TEOS, TMAP/TEOS) to 109° (C18-TMS/C8-TES/TEOS), depending on the functional group (see Table 2). The xerogels C8-TES/TEOS, C18-TMS/TEOS, TFP/C8-TES/TEOS, and C3TMS/TEOS have particularly high contact angles. The xerogels with aminoalkyl groups, in particular, show higher surface energies. The settlement of zoospores of *U. linza*, which was used to study marine fouling in the laboratory, increased with the static water contact angle in all xerogels of this study (the more hydrophobic, the higher the settlement; see [16], Figure 3a), which is in line with the trend of previous studies on *U. linza* [41,42,48,49,51]. To study fouling release properties, the colonized surfaces were cleaned, and the water jet pressure needed to remove the spore biomass was determined (see [16], Figure 4b). For the fluorocarbon/hydrocarbon-modified xerogels, spore removal correlated with increasing static water contact angle values and decreasing surface energy contributions. The more hydrophobic the layers are, the lower the needed water pressure and the easier the spores can be removed. The series of aminopropyl-modified xerogels did not follow these trends in spore removal and may reflect differences in the chemical reactivity of the various amino functionalities with the spore adhesives [16].

**Figure 8:**
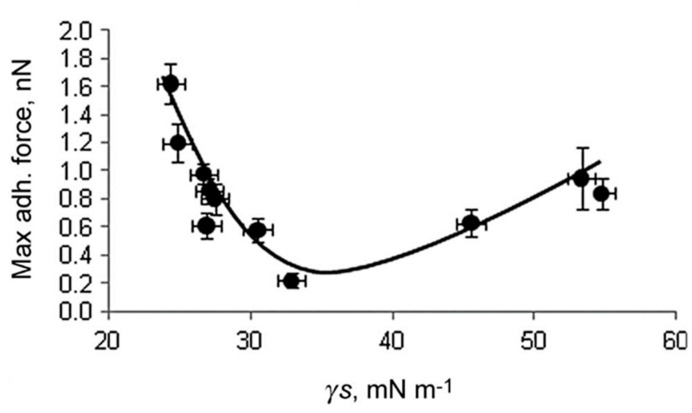
Maximum adhesion force of the BSA to the xerogel surface as a function of the critical surface tension. The measured data show a comparable course to the Baier curve (each value is the average of five repeated measurements) [20].

Finlay et al. used the same sol–gel coating systems as Bennet et al. [16,20]. The measured water contact angles (WCA) ranged from 35° (APTES/TEOS) to 105° (C18-TMS/C8-TES/TEOS), as in Bennet et al. Low contact angles were also measured for all other sol–gel layers with aminoalkyl groups. The adhesion of the samples was tested using *bovine serum albumin* (BSA). The adhesion of the BSA on all xerogels (0.22–1.62 nN) is significantly lower than on the PDMSE reference (3.5 nN). The adhesion values of the tested sol–gel coatings correlate with the surface tension and surface energy values and show a similar shape to the Baier curve (see Figure 8). The data show a range with the minimum BSA adhesion to the sol–gel coatings around 20–25 mN m^−1^ for the surface tension and about 30–35 mN m^−1^ for the surface energy. The lowest adhesion of the BSA was recorded on the PH/TEOS layer (0.22 nN) [20].

Furthermore, the adhesion of the diatoms *Navicula perminuta* to the different layers was tested. The initial adhesion to the different xerogels was comparable. However, the adhering cells were easier to remove from the hydrophilic xerogel surfaces by hydrodynamic shear, with a correlation between the percentage removal and the contact angle or surface tension for all coatings (see [20], Figure 4a). This corresponds to the studies of Finlay et al. and Schilp et al., who already observed that the proteins of diatoms are more easily detached from hydrophilic surfaces [41,42]. With the APTES/TEOS, DMAP/TEOS, and TMAP/TEOS coatings, the removal of diatoms was significantly higher than with the PDMSE standard (verified by statistical tests). Barnacle larvae (*B. amphitrite*) were used as the third adhesive species in this study. A significant difference between the coatings was already observed in the colonization of the cypris larvae. Settlement on the C18-TMS/C8-TES/TEOS, C8-TES/TEOS, C3/TEOS, TFP/TEOS, and TFP/PH/TEOS xerogel surfaces was reduced compared with the glass and polystyrene references. The colonization of *B. amphirite* on the C8-TES-TEOS xerogel (17 ± 5% fouling) was significantly lower than on the PDMSE reference (55 ± 17% fouling). The percentage of fouling correlates with the contact angle and the surface tension. The greater the contact angle, the lower the colonization by barnacle larvae. This corresponds to the behavior of *B. amphitrite* in previous studies (preferential attachment to hydrophilic surfaces) [43,44,45]. Based on their results, the authors suggested choosing a surface with medium wettability/surface energy, which is neither ideal for micro- nor macrofoulers but reduces the adhesion of both types of fouling to a certain extent [20].

Similar layer systems to Bennet et al. and Finlay et al. with the same or comparable chemicals and molar ratios were presented by Evariste et al. [16,20,31]. The study also used a TEOS xerogel with various siloxanes, such as fluorocarbon (TFP, TDF), aminopropyl (APTES, MAP, DMAP), and hydrocarbon groups (PH, C8-TES, C18-TMS). In addition to the green alga *U. linza*, which is frequently used in the literature, the adhesion of the brown alga *Ectocarpus crouaniorum* was also studied. The surface properties of the coating can change as a result of immersion. For this reason, the contact angles were measured in this study before and after immersion in artificial seawater (ASW). Pre-immersion WCAs in ASW were between 47° (MAP/TEOS)–108° (C18-TMS/C8-TES/TEOS). High contact angle values were determined for the C8-TES/C18-TMS/TEOS, C8-TES/TEOS, TDF/TEOS, and TFP/TEOS systems, whose surface energies are close to the Baier minimum before immersion. Like Bennet et al. and Finlay et al., the highest surface energies were obtained for the coatings with aminoalkyl groups. Immersion reduces the contact angles of all coatings by approximately 15°–25°, resulting in a lower variance of contact angles of 31°–91°. Similarly, the values of the surface energy (γ_S_) increased by 5–15 mN m^−1^ for all coatings. The two fluorinated xerogels (TFP/TEOS and TDF/TEOS) showed the largest percentage increase in surface energy after immersion in ASW of 53 and 118%, respectively. After about two to three weeks of drying, the contact angles corresponded to the values before immersion in ASW. The surface energy of the xerogels with aminoalkyl groups and the C18-TMS/C8-TES/TEOS xerogel increased by 20% after immersion. In the tests with *Ectocarpus crouaniorum*, the adhesion of the algae to the aminoalkylated coatings was significantly higher, and the percentage removal was the lowest compared with the other coatings. For the other xerogels, a weak adhesion of the algae was observed (80–95% removal), with only slight differences. In contrast to Bennet et al., no systematic influence of surface energy or contact angle could be identified for the fluorocarbon/hydrocarbon-modified xerogels. (see [31], Figure 3c) [31].

**Figure 9:**
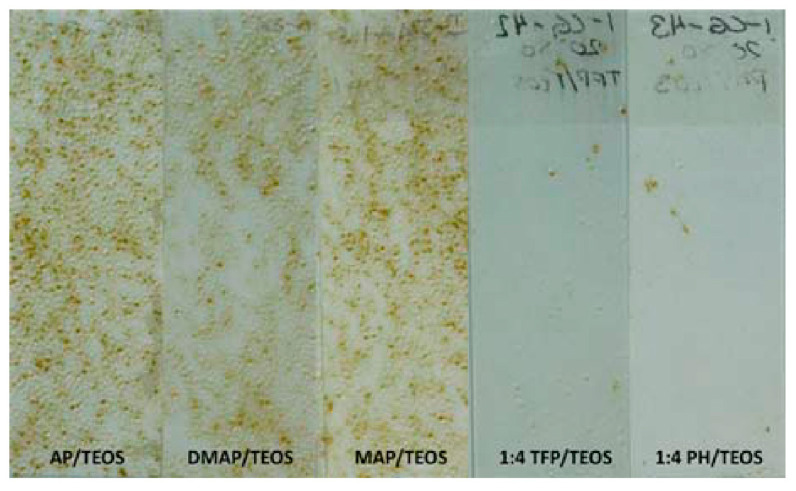
Photos of the coated samples after immersion showing the adhering algae growth (*E. crouaniorum*) after exposure to shear stress (8 Pa). For the xerogels with aminoalkylsilanes (APTES/TEOS, MAP/TEOS, and DMAP/TEOS), the proportion of bound biomass is significantly higher [31].

To determine the cause of the differing fouling release rate results for the aminoalkylated coatings, further analytical tests were performed. Using AFM, a surface roughness of 1 nm or less was measured for the coatings. This low roughness indicates no significant influence of this parameter. This and the results of the biological adhesion experiment suggest that surface charge may have a stronger influence on adhesion than wettability. Charged ammonium groups (experiments were performed at pH = 8.1) were confirmed on the surface of the aminoalkylated coatings with XPS after immersion in ASW. Subsequently, Evariste et al. developed a second series of coatings with similar surface topographies and surface energies (25.9–54.1 mN m^−1^) after immersion in ASW. The precursors of these coatings are siloxanes with side chains that were either neutral or contain side chains with amines that can be protonated in ASW to generate a positive charge. Again, adhesion tests were performed with *Ectocarpus crouaniorum*. The xerogels with aminoalkylsilanes (APTES/TEOS, MAP/TEOS, and DMAP/TEOS) had significantly less unbound biomass at the start of the experiment compared with the other coatings. This suggests that the adhesion of the algae to these coatings is already higher at the beginning. The bound biomass is also significantly higher after the incubation period (six days), and the removal rates are also very low (see Figure 9). The TFP/TEOS coating had the lowest bound biomass, and the removal rate (shear stress 8 Pa) of *E. crouaniorum* was the highest with PH/TEOS (90%). In the adhesion tests with the green algae *U. Linza*, a higher shear stress (33 Pa) was needed to remove the coating compared with *E. crouaniorum.* As in the experiments with the brown algae, the algal sporelings adhered less strongly to the surfaces with uncharged side chains, although all coatings had similar surface energy. Nevertheless, only removal rates of a maximum of 41% were achieved. This is due to the stronger adhesion of *U. Linza*. Electrostatic interactions between the positively charged aminoalkylsilanes on the coating surface and biological polymers may be a possible cause of the increased adhesion to these surfaces. Petrone et al. have already demonstrated for the spores of the brown algae *Undaria pinnatifida* that their biological adhesive consists of anionic polysaccharides [61]. The layers without positively charged side chains on the siloxanes tended to have a negative net charge. Due to the incomplete condensation reaction, free silanol functionalities (≡SiOH) can remain on the xerogel surface [62,63]. The pKa value of the silanol group (pKa ≈ 3) is similar to the carboxylic acid functionality (pKa ≈ 4) and is ionized in seawater (pH = 8.1 in these experiments), resulting in the formation of a net negative charge (of ≡SiO^−^) [31].

Sfameni et al. studied the antifouling and fouling release properties of hybrid coatings made from GPTMS and APTES in a molar ratio of 2°:°1 in ethanol. The mixture was stirred at room temperature for 24 h. The acid used for catalysis of the hydrolysis and condensation of the used alkoxysilanes was not given. After application and curing at 180 °C, it results in the formation of xerogel coatings. FT-IR analysis shows the successful formation of a silica network and the formation of an organic network via the polymerization of the epoxy and amino groups to form a hybrid network. Static contact angle measurements revealed slight hydrophilicity with a Wenzel water contact angle of 81.84° ± 0.85°. Other experiments included the addition 0.5 ω% of fluorinated compounds, namely TFP and F16, to the GPTMS/APTES/Ethanol mixture. After curing, these coatings exhibited even greater hydrophilicity with Wenzel water contact angles as low as 75.8°. Antifouling and fouling release properties were evaluated through diatom and bacteria settlement and adhesion essays and by exposure to fresh seawater. In comparison with the fluorine-free coatings, the coatings with fluorinated side chains showed higher diatom and bacteria removal rates [64].

Barletta et al. designed sol–gel-based coatings with amphiphilic surfaces to achieve antifouling and fouling release properties by introducing domains of highly hydrophilic surfaces as well as hydrophobic surfaces. Therefore, a low surface energy is achieved without losing the beneficial protein release properties of hydrophilic surfaces. Furthermore, the mosaic-like surface structure produced by the amphiphilic coating inhibits the adaptation of several organisms to the surface. The sol–gel process enables the introduction of hydrophilic groups, such as secondary amines, combined with hydrophobic groups, such as long-chain alkanes, through the substituents of the precursors. The authors used 1H,1H,2H,2H-perfluorooctyltriethoxysilane to introduce a highly hydrophobic substituent. As hydrophilic moiety, the commercial polyethylene glycol-functionalized alkoxysilane “Dynasylan 4150” was used. To synthesize the coatings, the components, as well as TEOS, were pre-hydrolyzed with hydrochloric acid as a catalyst to produce silanols. By introducing a hydroxy-rich siloxane (“Silikotop E901”) to the reaction mixture, grafting was achieved, yielding hydrophilic and hydrophobic resins. The amphiphilic coating solution was produced by mixing hydrophilic/hydrophobic resins in varying ratios and introducing the isocyanate-based hardener “Desmodur 3900”. The structure of the resulting coatings was analyzed in detail by IR spectroscopy, and the amphiphilic properties were analyzed by contact angle measurements. To evaluate the antifouling properties of the coatings, egg whites as a natural protein probe were applied to the samples. The amphiphilic coatings showed little to no adhesion of the egg white since it was removed by simple shaking [65].

### 3.2. Antifouling Coatings with Acrylates

Hamulić et al. examined six siloxane-based hybrid sol–gel systems with acrylate monomers on structured steel (S355), as it is often used for marine applications [66,67]. The alkyl chain length in the acrylate monomer varied from methyl methacrylate (MMA) to dodecyl methacrylate (DMA). TEOS was pre-hydrolyzed for one of the sols. The second sol consists of 3-(trimethoxysilyl)propyl methacrylate (MAPTMS), benzoyl peroxide, and the respective acrylate. The acrylate xerogel was obtained by mixing the two sols. The longer alkyl chain in the sol–gel structure is expected to reduce the wettability due to the hydrophobic alkyl groups and thus improve the fouling release properties of the coating. However, Hamulić et al. were unsuccessful in confirming this theory experimentally, as the coatings with ethyl methacrylate (EMA) and butyl methacrylate (BMA) in particular exhibited corrosion-reducing and antifouling properties. When measuring the contact angles, the WCA for the sol–gel layers with MMA (WCA_MMA_ = 61°) increased almost linearly to butyl methacrylate (WCA_BMA_ = 78°). With longer alkyl chains (WCA_DMA_ = 69°), the WCA decreases again due to the inhomogeneous mixing of the two sols. To determine the anticorrosive properties, a salt spray chamber test was used, and the coated samples were cross-cut with a blade to observe the effect of possible damage. A bare steel sample was used as a reference. This corroded within an hour on the first day. In the first nine days, the steel plates coated with the sol–gel layers with DMA, octyl methacrylate (OMA), and hexyl methacrylate (HMA) continued to corrode. The mildest corrosion progression was observed after 15 days for the coatings with EMA and BMA. The authors attributed this to the incomplete polymerization and condensation of inorganic and organic phases in the coatings with longer chains and the resulting increased porosity [66,68]. Field tests were performed in salt water (Adriatic Sea 38% salinity; Slovenia) and in freshwater (river Ižica, Slovenia). The samples were placed at a depth of approx. 1 m in plastic frames. The coated panels were inspected monthly, and the fouling was checked. The antifouling properties of the coatings with longer alkyl groups (OMA and DMA) could not be determined in salt water on steel because the surface was completely rusted after two months. After four months of immersion, the other coated panels were almost completely covered with *bryozoa* and *polychaeta*. The samples were then cleaned in an ultrasonic bath. All adhering contamination was removed after just one minute. Presumably, the contamination was removed directly during the movement of a boat. After cleaning, there are areas on the plates that reflect strongly. Despite the growth of marine organisms, no biocorrosion occurred. The ethyl- and butyl-based derivatives showed the best overall protection against biofouling and corrosion (mainly only from the edges of the panels) in salt water. The best results in terms of corrosion and biofouling after immersion in river water (five months) were obtained for the coatings with EMA, BMA, and HMA [67].

In addition, the toxicity of the coatings was tested by accelerated immersion tests in distilled water (heat, UV, ultrasonic bath). Samples of the surrounding liquid were taken regularly, and toxicity tests were performed with the water flea *Daphnia magna* and the green alga *Desmodesmus subspicatus*. The toxicity was lower than with the tested commercial biocide-containing coating (Seajet 034 Emperor) and decreased significantly after two weeks [67].

### 3.3. Combination of Fouling Release Layers with In Situ-Generated Antifouling Species

The use of fouling release coatings to control biofouling is limited by the fact that the fouling is only removed in the presence of hydrodynamic shear or by regular cleaning [22,30,59,69]. Coatings with the ability to generate substances with antifouling properties in situ to minimize the adhesion of fouling organisms by reagents naturally present in seawater or the influence of light energy represent an approach to combine the advantages of antifouling and fouling release coatings [18,32,70]. The advantage over conventional biocide-free fouling release coatings is that an antifouling effect is present even when the ship is stationary [71]. Ciriminna et al. developed a sol–gel coating that combines foul-release properties (C8-TES, C18-TMS, TEOS) and photocatalytic activity of Bi_2_WO_6_ (see Chapter 4. *Active surface catalysis for Antifouling properties on sol–gel-modified Surfaces*) [71].

An antifouling/fouling release layer composed of TEOS, C8-TES, and titanium tetraisopropoxide (TTIP) catalyzes in situ the oxidation of halide salts in seawater with hydrogen peroxide to form hypohalogenic acids [70,72]. The acids show a proven biocidal effect [73,74]. Hydrogen peroxide occurs in the open ocean in concentrations of up to 0.2 μM; in harbors fed by rainwater or runoff, the concentrations are significantly higher (up to 50 μM) [75,76,77]. As previously described [15,59], the incorporation of C8-TES into the xerogel formulations significantly lowers the surface energy (γ_S_) of the coating and results in a significant increase in contact angles (WCA_TEOS_ = 44°, WCA_C8-TES/TEOS_ = 103°) [70]. Replacing TEOS in the coatings with 20 mol% TTIP only had a small effect on the relative values of γ_S_ and the WCAs (WCA_C8-TES/TEOS/TTIP_ = 99°). After the immersion tests for 24 h in ASW, a reduction in the static WCAs (e.g., WCATEOS, immersed = 31°, WCA_C8-TES/TEOS_, immersed = 99°) was recorded for all four coatings tested. The change in surface energy and contact angle was significant compared with the respective values before immersion. The surface of the TTIP/C8-TES/TEOS coating was characterized by SEM; the characterization showed a uniform layer without cracks. This demonstrated that the organochalcogen compound does not change the surface structure and therefore does not reduce the fouling release properties. The antifouling properties of the xerogels could be improved by the addition of TTIP in the presence of hydrogen peroxide, which was confirmed by the study of zoospores and sporelings of *U. linza*. In the presence of H_2_O_2_ (50 μM), colonization was reduced by 11% on the C8-TES/TEOS coating and by 53% on the TTIP/TEOS coating. In the absence of hydrogen peroxide, no significant difference in the antifouling effect of the TTIP/C8-TES/TEOS compared with the C8-TES/TEOS xerogel was observed [70].

### 3.4. Fouling-Resistant Coatings with PEG or Zwitterionic Groups

The addition of hydrophilic PEG chains will increase the surface hydrophilicity, thus enhancing the antifouling performance. However, this approach is potentially problematic due to the swelling induced by the hydration layer on the surface, which can compromise the durability of the coating.

Shang et al. were able to circumvent this issue. The researchers created amphiphilic xerogel films from TEOS, C8 TES, C18 TMS, and poly(ethylene glycol) siloxane (PEG TMS) in varying compositions. In these films, surface swelling is suppressed by higher crosslinked density and smaller network aperture. After the film formation of the C18/C8/TEOS/PEG systems that incorporate both hydrophilic and hydrophobic groups, there are ripples detectable on the surface. A microphase segregation is observed in air due to the mutual repulsion between these groups. After immersion in water, these inhomogeneities are getting smaller, which proves a surface reorganization. The films display a worm-like nanostructure (around 2 nm deep) after submerging. The addition of C18 groups facilitates the emigration of PEG chains to the coating/air surface, while film formation enables the formation of the hydration layer later on. Furthermore, they help with the reorganization of the PEG groups on the surface when submerged. The addition of C18 TMS and PEG TMS shows significant improvements in protein absorption and bacterial attachment tests compared with C8 TES-doped TEOS films. In addition, they synergistically improve the antifouling properties. The contact angle measurements for the different sol–gel compositions are listed in Table 2 [78].

Chen et al. prepared a fouling-release hybrid coating starting from a telomere with amphiphilic side chains [79]. Which was synthesized from 3-(mercaptopropyl)triethoxysilane (MPTES), dodecafluoroheptyl methacrylate (DFMA) and poly(ethylenglycol) methyl ether methacrylate (PEGMA) in a molar ratio of 1:2:2 (telomer 1) in the presence of AIBN (Azobisisobutyronitrile) by radical polymerization. To study the influence of the individual reactants, alternative telomeres were synthesized without DFMA (1:4 MPTES/PEGMA—telomere 2) and without PEGMA (1:4 MPTES/DFMA—telomere 3). These telomeres were then reacted with C8-TES and TEOS using a sol–gel process. PEG is already widely used as a common antifouling agent, which reduces the adhesion of bacteria through a hydration layer (fouling-resistant layers) [79,80]. The coatings are transparent and characterized by good substrate adhesion and high hardness. Hardness is important in order to effectively prevent mechanical damage and thus also reduce the settlement of fouling. The surface energy of all hybrid coatings in this study lies between 17 and 27 mN m^−1^ and is therefore close to the Baier minimum. The sol–gel coating with the telomer without DFMA has a higher surface energy value of 27 mN m^−1^ due to the higher proportion of PEG groups (high SE). The coatings without PEGMA have the lowest surface energy of 14 mN m^−1^, which can be attributed to the high fluorine group content of DFMA. It can also be observed that the surface energy decreases with increasing telomere content [79].

In addition, immersion tests were performed in ASW. After immersion in ASW for three days, a marked decrease in the contact angle was observed for the layers with telomere 1 (with DFMA, PEGMA, and MPTES) (see [79] Figure 4). The higher the telomere content, the lower the contact angles (minimum 80°). This is caused by the accumulation of PEG on the coating surface. The telomer migrates to the surface as a result of the presence of DFMA. The coatings with the alternative telomeres without DFMA or without PEGMA showed no significant change in the contact angle in the immersion tests in ASW. The coatings without DFMA (telomere 2) and correspondingly high PEGMA content showed a higher contact angle than the immersed coatings with telomere 1, although telomere 2 contains a higher proportion of PEG groups. This shows that the PEGMA in telomere 2 tends to remain in the bulk because there is no DFMA in the layer, which migrates to the surface [79].

To analyze the antifouling properties against marine fouling, antibacterial tests (marine bacterium *Pseudomonas* sp.), biofilm formation (*Pseudomonas* sp.), and diatom density (*N. incerta*) were determined. In the antibacterial tests, the coating without telomere 1 and the layer with telomere 2 (without PEGMA) were completely covered with bacteria, which indicates that these layers have no antibacterial effect. In contrast, the antibacterial properties of the coatings increase with increasing telomere content (telomere with DFMA, PEGMA, and MPTES). In the coating with the highest telomere content, there is almost no bacterial growth. This is due to the accumulation of PEGMA on the surface as a result of the migration of DFMA and thus of the entire telomere 1 (see description of SE). The adhesive strength of the coatings produced was also tested. All coatings produced in the study achieved a higher adhesion (>0.7 MPa) to epoxy resin glass fiber sheets compared with PDMS. The telomeres with PEG were shown a higher adhesion value (1.5 MPa), as the PEG makes the coating tougher. The layers with PEG also showed an increase in flexibility in the bending test on PET (Polyethylene terephthalate) films. The addition of the telomer with DFMA, PEGMA, and MPTES resulted in a significant increase in hardness to 57 MPa, which is optimal for future application. The surface of the coating is therefore more resistant to damage [79].

**Figure 10:**
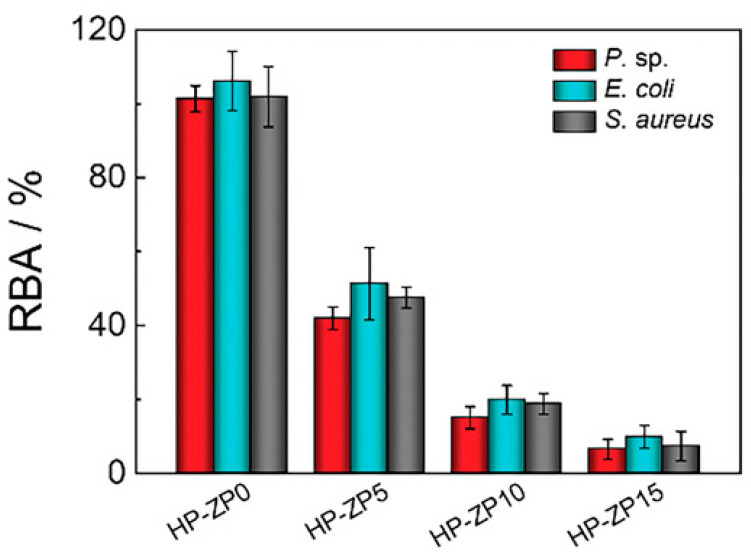
Relative bacterial adhesion (RBA) to the different xerogels. The higher the telomere content (HP-ZPx), the lower the adhesion of the tested bacterial cultures [81].

Chen et al. [81] presented a highly transparent antifouling coating (HP-ZPx) with fouling-resistant zwitterionic epoxy–zirconium particles (ZPx was prepared by the reaction of tetrapropyl zirconate (TPOZ) with GPTMS and zwitterionic sulfobetaine silane (SBSi)) and an amine-terminated hyperbranched polysiloxane. The hyperbranched siloxane (HPSi) with terminal amines is prepared by the hydrolysis of APTES. The zwitterionic particles are covalently bound to the HPSi. This leads to a high hardness and good substrate adhesion of the layers. The highly branched polysiloxane improves the mechanical properties (e.g., elastic deformability) of the coating compared with a linear polysiloxane, as it has more interaction and reaction sites [81,82,83]. In the study, the mass ratio of the sulfobetaine silane to TPOZ and GPTMS was varied (ZPx, x = 0–15, x is the mass ratio SBSi in the TPOZ and GPTMS). During the characterization of the coatings, it was shown that the hardness decreases with increasing amounts of the zwitterionic silane because the zwitterionic groups act as plasticizers. This is also reflected in the results of the abrasion test, as the wear resistance also decreases with increasing SBSi content. The static WCA was determined in the study. The contact angle values reflect the hydrophilic character of the surface. The addition of the zwitterionic component increases the hydrophilicity (WCA_HP-ZP0_ = 83.4°; WCA_HP-ZP15_ = 76.0°). The dynamic contact angles of the layers were also measured. The receding WCAs (CA_HP-ZP0_ = 45.4°; CA_HP-ZP15_ = 21.3°) varied more than the static water contact angles, as these contact angles are more dependent on the hydrophilicity of the coating. These results suggest that the zwitterionic groups in this system migrate to the surface of the coating and thus increase the hydrophilicity. The zwitterionic groups form an electrostatically induced hydration layer at the interface between the coating and the surrounding medium. This hydration layer forms both a physical and energetic barrier of strongly bound water molecules, which reduces the settlement of organisms [84,85,86]. The antibacterial properties of the coating were tested with three different bacterial strains (*Pseudomonas* sp., *Escherichia coli*, *Staphylococcus aureus*) that occur in aquatic ecosystems or in daily environments. The antibacterial character of the coating with zwitterionic particles was confirmed, as bacterial adhesion of all tested bacterial strains decreases with increasing proportion of zwitterionic groups (HP-ZP15~5% bacterial adhesion compared with bare substrate see Figure 10) [81].

**Figure 11:**
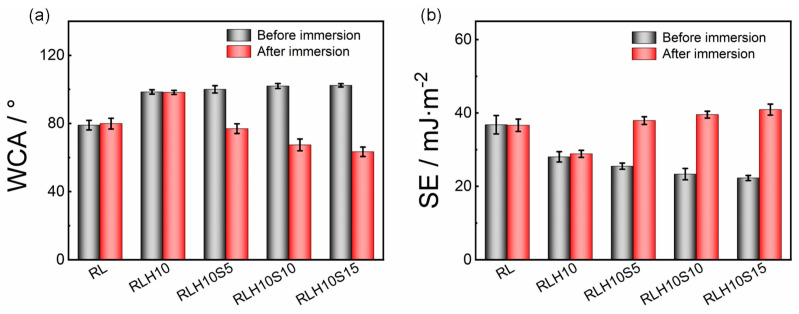
Plot of the static water contact angles (**a**) and the surface energy (**b**) of the natural varnish (RL), the natural varnish with HPSi (RLH10), and the layers to which the telomer (RLH10Sx) was added before and after immersion in water (24 h) [80].

In a further study, Chen et al. [80] synthesized a xerogel layer starting from the HPSi, which was produced by the hydrolysis of APTES [81] and the amphiphilic telomer of DFMA, PEGMA, and MPTES (see [79]) and a natural varnish (RL) via the sol–gel reaction. The telomer content (S-FP 0–15 ω%) in the layers was varied to study the influence of this component. The reaction is based on the alcoholysis between the hydroxyl group of the urushiol (a component of the natural varnish) and the alkoxy group of the organic silanes, as well as the sol–gel reaction of the siloxanes, both of which can promote the polymerization of urushiol. In contrast to pure natural coatings, which require a long drying time, this is shortened by the addition of HPSi. The HPSi also improves the mechanical properties of the coating, such as the surface hardness. The antibacterial properties of the coating were tested with the bacterial strains *Pseudomonas* sp., *Escherichia coli,* and *Staphylococcus aureus*. Bacterial growth is slightly reduced by coating with the natural varnish or the natural varnish with polysiloxane compared with the blank. The addition of the telomer, on the other hand, results in a strong reduction in fouling, which is due to the PEG. The antifouling polymer prevents the adsorption of bacteria. The commercially available natural varnish has a WCA of 79°. The addition of the hyperbranched polysiloxane (10 ω% HPSi) increases the hydrophobic character (WCA = 98°). The layers with added telomer show only a slightly higher contact angle depending on the telomer content (100°–105°). The samples were stored in water for 24 h, and the contact angle was measured again. The samples without telomer showed no change in wetting properties. The coatings with telomer showed lower contact angles (approx. 75°–63°) after immersion, which can be attributed to the highly hydrophilic PEG segment (see Figure 11). As already shown by Chen et al. [79], the telomere migrates to the surface due to the presence of DFMA and can thus better develop the antifouling effect [80].

Tan et al. developed a sol–gel-based, hard coating of TEOS and organoalkoxysilanes with zwitterionic and isothiazolinone groups. This coating is to be used specifically in underwater robotic cleaning technology. Two innovative silanes were used for the synthesis: (N-Methoxyacylethyl)-3-aminopropyltriethoxysilane (MAPS) and 2-(2-Hydroxy-3-(3-(trimethoxysilyl)propoxy)propyl)benzo[d]isothiazol-3(2H)-one (BITS). MAPS forms zwitterionic structures through hydrolysis in neutral or alkaline solutions. BITS, on the other hand, acts as an environmentally friendly antibacterial agent, as the activated N-S bonds interfere with cellular structures and exert an antimicrobial effect [87].

Three series of hybrid antifouling coatings were prepared: Z-χ (MAPS/TEOS) with zwitterionic groups, A-χ (BITS/TEOS) with antibacterial groups and S-χ (MAPS/BITS/TEOS) with a combination of zwitterionic and antibacterial groups to investigate synergistic effects. The silane contents were also varied in all three coating series. One of the authors’ aims was to produce a coating with a high hardness. A maximum value of 5 H was achieved with the pencil hardness measurement; a higher TEOS content led to increased hardness due to the increased cross-linking of the Q groups [87].

Laboratory and field tests showed that an increasing TEOS content also improved the antifouling (AF) performance of all three coating series. A protein adsorption test with fluorescein-labeled *bovine serum albumin* (BSA) revealed that A-χ showed the highest protein adsorption at the same TEOS content, while Z-χ and S-χ showed lower values. The protein resistance of the MAPS-containing layers resulted from a hydration layer that acted as a barrier. In the bacterial adhesion test (*Pseudomonas marina*), the BITS-containing layers (A-χ, S-χ) proved to be more resistant to *P. marina*. Both in the mussel colonization test (*Mytilus coruscus*) and in the one-month marine field test in the East China Sea (Pacific Ocean, Hangzhou Bay), the S-χ coating showed the best antifouling effect. In the field test, the S-1 coating (BITS [12.5 mmol]/MAPS [12.5 mmol]/TEOS [25 mmol]) was able to reduce the diatom density by around 54% compared with uncoated glass slides after seven days of immersion. The combination of MAPS and BITS combines the repulsive hydration power of MAPS, while BITS effectively eliminates the remaining bacteria. Contact angle measurements showed that the hydrophilicity of the zwitterionic group in Z-χ was increased by hydrolysis in ASW, with the contact angle decreasing from 61°–75° to 14.5°–19°. S-χ showed a slower downward trend, while A-χ showed almost constant values over the entire period, which can be attributed to the absence of a hydrophilic component (see Table 2) [87].

In another study, Tan et al. enhanced the properties of a transparent PDMS coating by utilizing MAPS, which was co-hydrolyzed with TEOS (h-MAPS, with the weight ratio of MAPS to TEOS varied). The hydrolysis occurred under acidic conditions (0.01 M HCl) with anhydrous ethanol as the solvent. The final coatings were prepared by mixing h-MAPS with PDMS in different weight ratios, using THF as the solvent, and adding dibutyltin dilaurate (DBTDL) as a catalyst. As substrates, glass-fiber-reinforced epoxy resin, plastic Petri dishes, or 12-well cell culture plates were used. The cast coatings were cured under ambient conditions. If curing was incomplete after 5 days, the coatings were further heated to 80 °C for 4 h, followed by 120 °C for 1 h. In comparison with previous work using purely MAPS/TEOS sol–gel coatings, it was demonstrated that incorporating PDMS with h-MAPS significantly improved marine antifouling protection. The effectiveness was shown to increase with higher zwitterion-bearing silica sol (h-MAPS) content rather than with PDMS alone [88].

A paper by Sun et al. discusses the development of an eco-friendly, hard sol–gel coating (based on BITS) with a nonleaching antifoulant (based on KH580, SBMA, and HFBM) designed to prevent biofouling in static marine environments. By incorporating zwitterionic and hydrophobic nanodomains, the coating effectively inhibits microbial attachment and growth on its surface. It exhibits high hardness (6H pencil hardness) and stability, making it resilient to mechanical stress, which is especially useful for applications on ship propellers and hulls. The antifoulant molecules are permanently bound within the coating, preventing their release into the water, thus providing a sustainable and environmentally safe solution. Overall, this technology offers an effective combination of antiadhesion and fouling-release properties for marine applications [89].

### 3.5. Superhydrophobic and Superamphiophobic Coatings

**Figure 12:**
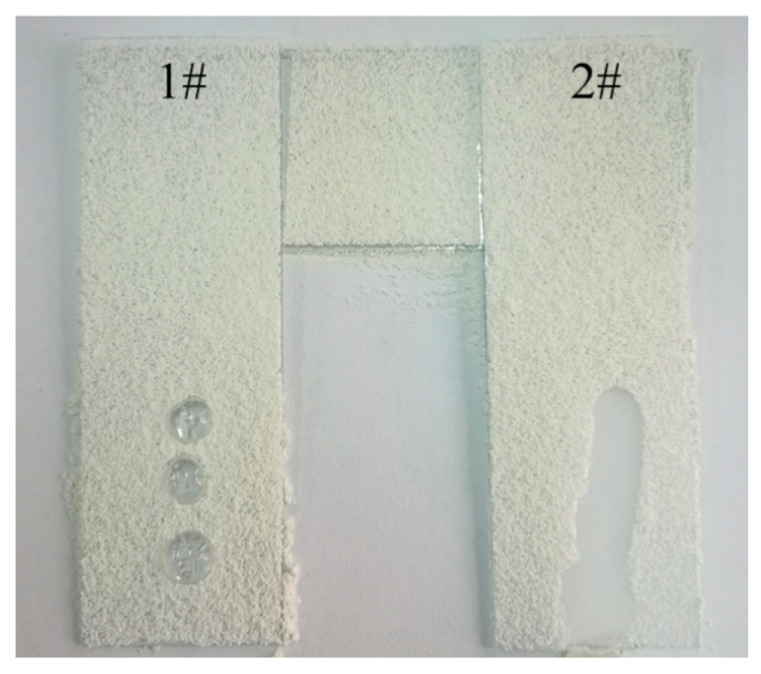
Photos of the uncoated (**#1**) and coated (**#2**) glass slides after adding chalk powder and applying water to demonstrate the self-cleaning properties of the superhydrophobic sol–gel layer (#2—water rolls off and removes the chalk) [92].

Feng et al. used TEOS and *n*-octyltrimethoxysilane (C8-TES) as precursors for a superhydrophobic transparent sol–gel coating. A surface is described as superhydrophobic if the contact angle is greater than 150° [90,91]. The superhydrophobicity in this system is caused by the synergistic effects of the rough nanometer-scale SiO_2_ structures (50 nm diameter nanoparticles) and the low surface energy hydrophobic alkyl group. The sol–gel method is one of the established ways to synthesize nanoparticles (homogeneous noncrystalline solids of high purity). The hydrolyzed C8-TES reacts with the hydrophilic SiO_2_ nanoparticles, which were synthesized by basic hydrolysis (NH_4_OH) and condensation reactions starting from TEOS. The layers were extensively characterized by contact angle analysis under different conditions. The static WCA is about 160°. The trapped air in the fractured hierarchical structures of the coating presumably prevents the water from adhering. The superhydrophobicity of the coating persists in the range of 0 °C–180 °C (for 6 h) and up to a pH value of 12 (for 24 h). At a pH of 14, the contact angle drops extremely sharply to 122.5°, and the coating partially peels off during immersion. The self-cleaning properties of the coating were confirmed experimentally. Chalk was placed on an uncoated (#1) and a coated (#2) glass slide, and then water was dripped onto the surface. On the slide with the superhydrophobic sol–gel layer, the water beads up and rinses off the chalk powder. In the case of the uncoated substrate, the drop remains on the substrate (see Figure 12) [92].

The xerogel layers can also be modified using superamphiphobic materials. Superamphiphobic materials are characterized by their self-cleaning, water- and oil-repellent properties, as evidenced by a water and oil contact angle >150° [93]. Special rough surface structures and substances with very low surface tension, such as fluorinated materials with -CF3- and -CF2-groups, are necessary to produce superamphiphobic coatings [94]. Chen et al. [93] produced a superamphiphobic fabric using a simple, timesaving, two-stage process. The Si component (TEOS) was deposited with a thiol (MPTES) in the presence of ammonia as a catalyst via a vapor–liquid–sol–gel reaction. Silicon dioxide particles (silica particles) with thiol groups were formed on the fabric by the hydrolysis and condensation reaction of TEOS with MPTES. Concurrently, the hydroxyl groups of the formed particles can condense with the hydroxyl groups of the tissue, which increases the adhesion to the tissue. The textile already exhibits superhydrophobic properties after this treatment. Afterward, the superamphiphobic fabric was generated by photoinitiated thiol–Ene click reaction between the thiol groups of the particles and the Ene group of the 1H,1H,2H,2H-heptadecafluorodecyl methacrylate (FMA). Chen et al. varied the TEOS/MPTES ratio in the range of 1:1–1:4. Increasing the TEOS/MPTES mass ratio could result in hierarchical and hollow layer, thus improving the hydrophobicity and oleophobicity of the fabric. The ratio of 1:3 shows the best superamphiphobic properties with a WCA of 158°. At a ratio of 1:4, numerous silica particles grew together and formed a more compact structure, resulting in a decrease in amphiphobicity as the Ene group had less opportunity to bind. The ammonia contact time was also varied between 5–20 min. At a treatment time of 20 min, the silica particles formed hierarchical and hollow structures, and the tissue exhibited WCAs of >150°. This results in an optimum TEOS/MPTES ratio of 1:3 and an ammonia exposure time of 20 min. A layer of air forms between the surrounding medium and the fabric, which inhibits/reduces wetting or fouling. It can therefore be said that the superamphiphobic fabric is not only suitable for self-cleaning but also an ideal material for antifouling properties [93].

Another approach to creating hydrophobic surfaces is described by Rodriguez et al. [95]. First, a hydrophobic aerogel or xerogel is produced. This is then ground and applied to an adhesive surface. MTMS was used to modify the tetramethoxysilane (TMOS)-based recipe. The methyl group cannot participate in the sol–gel process and creates chemical hydrophobicity in the material. The resulting aerogels and xerogels have very different morphologies with comparable chemical hydrophobicity. The average pore size of the xerogels (~4 nm) is smaller than that of the aerogels (10–15 nm). Both show increased surface areas with small amounts of added MTMS, but the addition of too much MTMS leads to a decrease in surface area (see [95] Figure 10). All surfaces coated with modified aerogels are superhydrophobic. The hydrophobicity of the xerogel-coated surfaces increases by increasing the amount of MTMS from hydrophilic at low levels (10–25%) to comparably superhydrophobic as the aerogels at high levels (around 75% MTMS) (see [95] Figure 9).

**Table 2:** Overview of the current status of papers on the subject of fouling-release and fouling-resist systems, including the contact angles (CA), surface energies (SFE), and tensions (STE) listed.

Structural Unit	Precursors	Ratio Precursors Molar (mole-%) * Mass (ω%) *^1^ Volume (V%) *^2^	WCA θ_Ws_°	SFE γ_S_ (mN m^−1^)	SFT *γ_C_* (mN m^−1^)	Special Contact Angles or Surface Energy	Reference
Reference	TEOS	---	44 ± 2	56 ± 2	---	CA (CH_2_I_2_): 46 ± 2° after immersion in ASW 24 h WCA: 31 ± 1° SFE: 63.9 ± 0.6 mN m^−1^ CA (CH_2_I_2_): 47 ± 1°	[70,72]
Alkyl chains	C3-TMS/TEOS	1:1 *	99 ± 1	27.5 ± 1.1	21.3 ± 0.1	---	[16]
		50:50 *	99 ± 1	---	---	---	[20]
	C8-TES/TEOS	40:60 *	102.8 ± 0.6	23.2 ± 0.2	---	CA (CH_2_I_2_): 69 ± 1° after immersion in ASW 24 h WCA: 99.4 ± 0.8° SFE: 26 ± 1 mN m^−1^ CA (CH_2_I_2_): 65 ± 1°	[70,72]
		1:1 *	104 ± 1	27.1 ± 0.3	21.3 ± 0.1	---	[16]
		50:50 *	104 ± 1	---	---	---	[20]
		50:50 *	100 ± 2	27.1 ± 0.3	21.3 ± 0.1	Surface roughness: 0.24 + 0.02 nm	[57]
		1:1 *	96 ± 6	25 ± 1	---	after immersion in ASW 24 h WCA: 80 ± 5° SFE: 35 ± 2 mN m^−1^	[31]
		1:1 *	---	---	---	---	[22]
		1:1 *	---	21.5	---	---	[33]
		1:1 *	---	---	---	---	[58]
	C8-TES/TEOS (*Alkaline catalysed*)	1:2 *^2^	159.5–163.1 ^(1)^	---	---	WSA: 4–5 ^(1)^ stability of the superhydrophobicity 0 °C–180 °C and pH 0–10	[92]
	C8-TES/TEOS/TTIP	40:40:20 *	99 ± 1	25.0 ± 0.1	---	CA (CH_2_I_2_): 66 ± 1° after immersion in ASW 24 h WCA: 94 ± 2° SFE: 27 ± 1 mN m^−1^ CA (CH_2_I_2_): 63 ± 1	[70,72]
	C18-TMS/C8-TES/TEOS	1:49:50 *	111.2 ± 0.2	24.8 ± 1.1	21.4 ± 0.1	Surface roughness: 1.15 + 0.04 nm	[57]
		1:49:50 *	---	---	---	---	[22,33]
		0,1:0,9:1 *	105 ± 1	24.6 ± 0.9	21.9 ± 0.3	---	[16]
		2:48:50 *	108.3 ± 0.9	22.8 ± 1.1	---	Surface roughness: 0.67 + 0.03 nm	[57]
		3:47:50 *	102 ± 4	25.7 ± 2.1	22.4 ± 0.9	Surface roughness: 0.22 + 0.02 nm	[57]
		4:46:50 *	105 ± 2	22.8 ± 1.1	---	Surface roughness: 0.20 + 0.01 nm	[57]
		5:45:50 *	105 ± 1	---	---	---	[20]
		5:45:50 *	108.2 ± 0.9	24.6 ± 0.9	21.9 ± 0.3	---	[57]
		5:45:50 *	107.9 ± 0.7	22.0 ± 0.4	---	after immersion in ASW 24 h WCA: 91 ± 3° SFE: 27 ± 1 mN m^−1^	[31]
		1:49:50	104.6 ± 0.8	---	---	θw,re (°) = 84.1 ± 0.7; Hysteresis (°) = 20.5	[78]
	C18-TMS/C8-TES/TEOS + Bi_2_O_6_	1:49:50	---	---	---	---	[71]
	C8-TES/PEG-TMS/TEOS	49:2:50	100.7 ± 0.9	---	---	θw,re (°) = 76.9 ± 0.6; Hysteresis (°) = 23.8	[78]
		49:4:50	95.8 ± 1.0			θw,re (°) = 71.2 ± 0.4; Hysteresis (°) = 24.6	
		49:8:50	88.6 ± 1.8			θw,re (°) = 63.3 ± 2.0; Hysteresis (°) = 25.3	
	C18-TMS/C8-TES/PEG-TMS/TEOS	1:49:2:50	102.5 ± 0.5	---	---	θw,re (°) = 82.9 ± 0.4; Hysteresis (°) = 19.6	[78]
		1:49:4:50	101.8 ± 1.2			θw,re (°) = 80.8 ± 0.7; Hysteresis (°) = 21.0	
		1:49:6:50	99.7 ± 1.1			θw,re (°) = 77.2 ± 2.4; Hysteresis (°) = 22.5	
		1:49:8:50	89.4 ± 2.2			θw,re (°) = 65.1 ± 1.3; Hysteresis (°) = 24.3	
		1:49:10:50	85.8 ± 5.3			θw,re (°) = 53.9 ± 6.5; Hysteresis (°) = 31.9	
Fluorinated alkyl substances	TFP/TEOS	1:1 *	85 ± 1	26.9 ± 0.3	18.8 ± 0.1	---	[16]
		50:50 *	85 ± 1	---	---	---	[20]
		1:1 *	83 ± 1	25.7 ± 0.7	---	after immersion in ASW 24 h WCA: 64.2 ± 0.2° SFE: 39.3 ± 0.3 mN m^−1^	[31]
		1:4 *	83 ± 1	25.9 ± 0.9	---	after immersion in ASW 24 h WCA: 44 ± 9° SFE: 55 ± 6 mN m^−1^	
	TDF/TEOS	1:1 *	105 ± 3	12.4 ± 0.8	---	after immersion in ASW 24 h WCA: 83 ± 2° SFE: 27 ± 2 mN m^−1^	[31]
	GPTMS/APTES with TFP	2:1 * with 0.5 ω% TFP	81.84 ± 0.85 ^(x)^	---	---	---	[64]
	GPTMS/APTES with F16	2:1 * with 0.5 ω% F16	81.44 ± 0.85 ^(x)^	---	---	---	
	GPTMS/APTES with TFP/F16	2:1 * with 0.5 ω% TFP/F16 (1:1)	75.80 ± 0.95 ^(x)^	---	---	---	
	TDF/TEOS with PEG	50:50 * with 0.5 mol%	---	---	---	---	[22]
	TEOS/FPES	96:4	105–110	---	---	CA (*n*-Hexadecane): 63–67° after 100 wear cycles WCA: 56.0 ± 5.55° CA (*n*-Hexadecane): 33.3 ± 5.06° after 90 wear cycles µ = 0.131 ± 0.007	[96]
	TEOS/FPES with F-127	96:4 with 8 mol%	105–110	---	---	CA (*n*-Hexadecane): 63–67° after 100 wear cycles WCA: 91.8 ± 3.66° CA (*n*-Hexadecane): 49.8 ± 2.51° after 90 wear cycles µ = 0.014 ± 0.003	[96]
Aryl groups	PH/TEOS	1:1 *	90 ± 1 ^(2)^ 74 ± 1 ^(3)^	32.9 ± 0.5 40.0 ± 0.2	24.5 ± 1.6 35.2 ± 0.2	--- ---	[16]
		50:50 *	74 ± 1	---	---	---	[20]
		1:1 *	80.4 ±0.6	38.2 ± 0.3	---	after immersion in ASW 24 h WCA: 54 ± 2° SFE: 53 ± 1 mN m^−1^	[31]
		1:4 *	81 ± 1	36.8 ± 0.2	---	after immersion in ASW 24 h WCA: 59 ± 2° SFE: 50 ± 1 mN m^−1^	
Amino/Ammonium groups	APTES/TEOS	1:9 *	57 ± 1	53.3 ± 0.2	34.2 ± 0.1	---	[16]
		10:90 *	35 ± 1	---	---	---	[20]
		1:9 *	56 ± 1	52 ± 1	---	after immersion in ASW 24 h WCA: 39 ± 2° SFE: 62.2 ± 0.4 mN m^−1^	[31]
	APTES/GPTMS	1:2 *	81.84 ± 0.85 ^(x)^	---	---	---	[64]
	MAP/TEOS	1:9 *	57 ± 1	47.9 ± 0.7	25.2 ± 0.7	---	[16]
		10:90 *	57 ± 1	---	---	---	[20]
		1:9 *	49 ± 1 47 ± 2 ^(4)^	53.5 ± 0.8 54 ± 1 ^(4)^	---	after immersion in ASW 24 h WCA: 32.6 ± 0.8° WCA: 33 ± 3° ^(4)^ SFE: 63.6 ± 0.3 mN m^−1^ SFE: 66 ± 2 mN m^−1 (4)^	[31]
	DMAP/TEOS	1:9 *	35 ± 1	54.7 ± 2.7	32.2 ± 2.0	---	[16]
		10:90 *	42 ± 1	---	---	---	[20]
		1:9 *	50 ± 3 48 ± 3 ^(4)^	53 ± 2 53 ± 2 ^(4)^	---	after immersion in ASW 24 h WCA: 31 ± 3° WCA: 24 ± 6° ^(4)^ SFE: 64 ± 2 mN m^−1^ SFE: 69 ± 3 mN m^−1 (4)^	[31]
	TMAP/TEOS	1:9 *	35 ± 1	---	---	---	[16]
		10:90 *	54 ± 1	45.5 ± 0.1	29.7 ± 0.1	---	[20]
	BITS	---	123	---	---	UWOCA: 123° After immersion in ASW for 15 days WCA: 119.5° UWOCA: 119.5°	[87]
	BITS/TEOS	44.8:5.6–25.0:25.0	60–69	---	---	UWOCA: 116°–123° After immersion in ASW for 15 days WCA: 60°–69 UWOCA: 120°–124°	
Acrylate	MAPTMS/TEOS/MMA	1:2:8 *	61 ± 2	---	---	---	[67]
	MAPTMS/TEOS/EMA	1:2:8 *	70 ± 1	---	---	---	[67]
	MAPTMS/TEOS/BMA	1:2:8 *	78 ± 1	---	---	---	[67]
	MAPTMS/TEOS/HMA	1:2:8 *	69 ± 1	---	---	---	[67]
	MAPTMS/TEOS/OMA	1:2:8 *	69 ± 1	---	---	---	[67]
	MAPTMS/TEOS/DMA	1:2:8 *	69 ± 1	---	---	---	[67]
Various functional groups	TFP/C3-TMS/TEOS	1:1:2 *	92 ± 1	24.9 ± 0.6	20.3 ± 0.1	---	[16]
		25:25:50 *	92 ± 1	---	---	---	[20]
	TFP/C8-TES/TEOS	1:1:2 *	100 ± 1	24.4 ± 0.3	20.4 ± 0.3	---	[16]
		25:25:50 *	100 ± 1	---	---	---	[20]
	TFP/PH/TEOS	1:1:2 *	84 ± 1	26.7 ± 0.3	21.0 ± 0.2	---	[16]
		25:25:50 *	84 ± 1	---	---	---	[20]
	C8-TES/PH/TEOS	1:1:2 *	94 ± 1	30.5 ± 0.6	24.5 ± 0.5	---	[16]
		25:25:50 *	94 ± 1	---	---	---	[20]
	APTES/C8-TES/TEOS	1:9:9 *	---	---	---	---	[60]
	C18-TMS/TDF/TEOS	1:49:50 *	97.0 ± 1.1	17.3 ± 0.5	---	---	[58]
	C18-TMS/TDF/C8-TES/TEOS	1:1:48:50 *–1:24:25:50 *	98.9 ± 1.6–110.3 ± 0.7	6.1 ± 3.021.8 ± 2.8	11.5 ± 2.3–19.8 ± 0.5	---	[58]
	PDMS	all containing TBAF 0.3 ω% MTAcS 21 ω%	110 ± 3	---	22.4	HCA: 37 ± 1° WCA: 103 ± 1° ^(6)^ HCA: 30 ± 2° ^(6)^	[97]
	PDMS/PEOTMS	1:2–1:10	103–113	---	22.7–23.3	HCA: 35–37° WCA: 107–108 ^(6)^ HCA: 37° ^(6)^ γ_S_: 22.5–24.6 mN m^−1 (6)^	[97]
	PDMS/PFOTES	1:2–1:50	106–111	---	11.9–22.0	HCA: 40–76° WCA: 101–109° ^(6)^ HCA: 40–71° ^(6)^ γ_S_: 14.5–21.8 mN m^−1 (6)^	[97]
	PDMS/(PEOTMS/PFOTES)	1:(1:9–4:6)	101–106	---	22.9–23.8	HCA: 35–36° WCA: 108–109° ^(6)^ HCA: 35–36° ^(6)^ γ_S_: 22.7–23.0 mN m^−1 (6)^	[97]
Superamphiphobic coatings	TEOS/MTEO (FMA/HMPF)	1:1–4:1 *^1^	148–159	---	---	UOWCA: 161° UWOCA: 161° HCAs: 137–152° WSA: 2–6° HSA: 8–12°	[93]
Amphiphilic coatings	C8-TES/TEOS + *Telomer contains*: MTEO/DFMA/PEGMA *or* MTEO/PEGMA *or* MTEO/DFMA	1:1 * 1:2:2 * (0–15 ω%)	--- ~105	--- 17–27 ^(5)^	--- ---	after immersion in ASW 4 days ~80°–90° ^(5)^	[79]
		1:4 *	95	27	---	95°	
		1:4 *	~113	14	---	~113°	
	Natural Lacquer/hyperbranched siloxane (Hydrolyse von APTES) + *Telomer contains*: MTEO/DFMA/PEGMA	9:1 *^1^ 1:2:2 * (0–15 ω%)	98–105 ^(5)^	~22–28 ^(5)^	-	after immersion in water for 24 h WCA: ~98–63° ^(5)^ (decrease with Telomer) SFE: ~29–41 mN m^−1 (5)^ (increase with Telomer)	[80]
Zwitterionic layers (particle)	zwitterionic epoxy–zirconium particle GPTMS/TPOZ//SBSi hyperbranched siloxane Hydrolyse von APTES	Epoxy–zirconium particle (mass ratio of TPOZ and GPTMS to SBSi)				advancing WCAs: 88.1°–−78.1° receding WCA: 45.5°–21.3° UWOCA: 46.0°–85.6° After UV radiation 84.4° (1:0) 79.6° (10:1) Liquid resistance test in Hexane, xylene, dimethyl-sulfoxide, ethanol (24 h) 81.3°–83.8° (1:0) 77.3°–78.4° (10:1) Liquid resistance test in 0.1 M HCl (24 h) 71.4° (1:0) 64.3° (10:1) Liquid resistance test in 0.1 M NaOH (24 h) 53.8° (1:0) 51.9° (10:1)	[84]
		1:0 *^1^	83.4	---	---		
		5:1 *^1^	80.4	---	---		
		10:1 *^1^	78.5	---	---		
		15:1 *^1^	76.0	---	---		
	MAPS	---	75	---	---	UWOCA: 118°	[87]
	MAPS/TEOS	44.8:5.6–25.0:25.0 *	61–75	---	---	UWOCA: 108°–118° After immersion in ASW for 15 days WCA: 14.5°–19° UWOCA: 129°–134°	
	BITS/MAPS/TEOS	25.0:25.0:0–12.5:12.5:25.0 *	63–70	---	---	UWOCA: 109°–118° After immersion in ASW for 15 days WCA: 53°–62° UWOCA: 125°–130°	
PEGylated	TTIP/PEG	1:9–1:3	15–35	---	---	WCA 100% TTIP: 55° WCA 100% PEG: 11°	[98]
Polysaccharid	Chitosan/GPTMS/TMOS	1:4 * (GPTMS/TMOS)	---	30.39	---	advancing WCA: 73° receding WCA: 20° advancing HCA: 18° receding HCA: 8° SFE (Hexadecane): 26.14 mN m^−1^	[8]
	Chitosan/TMOS		---	35.20	---	advancing WCA: 67° receding WCA: 29° advancing HCA: 21° receding HCA: 6° SFE (Hexadecane): 25.67 mN m^−1^	[8]
	Chitosan/VTMS		---	28.84	---	advancing WCA: 75° receding WCA: 18° advancing HCA: 12° receding HCA: 9° SFE (Hexadecane): 13.28 mN m^−1^	[8]

* The sol/xerogel composition is designated in terms of the molar ratio of Si-containing precursors. Thus, a 1:1 PH/TEOS composition consists of 50 mole-% PH and 50 mole-% TEOS. ^1^ Depending on repetition of dip-coating (1–6 times). ^2^ Sol was stirred at ambient temperature for 48 h to give a more hydrophobic coating. ^3^ Sol was stirred for 7 days to give a more hydrophilic coating. ^x^ Wenzel’s contact angle calculated from width and height of a static drop. ^4^ Various measurement series. ^5^ Depending on the telomere content (variation in weight percentage of telomere/(TEOS-C8-TES)). ^6^ After immersion in deionized water for 14 days.

## 4. Active Surface Catalysis for Antifouling Properties on Sol–Gel-Modified Surfaces

To achieve antifouling properties on surfaces that are coated with a layer produced by the sol–gel process, the incorporation of an active component into the layer is one strategy. Most of the used catalysts share the mechanism that is responsible for the antifouling effect, which uses the photocatalytic cleavage of water into reactive products like reactive oxygen species (ROS). Scandura et al. used bismuth tungstate (Bi_2_WO_6_) nanoparticles encapsulated in organically modified silicate (ORMOSIL), which was synthesized by using the sol–gel process [99]. Bi_2_WO_6_ was chosen because of several reasons. Its narrow band gap of 2.8 eV allows absorption of visible light and therefore photocatalysis when exposed to sunlight is possible. The photocatalytic properties of Bi_2_WO_6_ have been investigated in various studies and are therefore well known [100,101]. Furthermore, it is a nontoxic material that can be easily manufactured by various methods like hydrothermal synthesis, solid-state synthesis, or the sol–gel method [101].

Scandura et al. used flower-shaped Bi_2_WO_6_, which was prepared using the hydrothermal method. These nanoparticles were incorporated into several TEOS/MTES-sols by adding them to the sol, which was sonicated and stirred afterward. The samples were radiated with simulated solar light in a defined volume of deionized water. After the irradiation, the water was examined to determine the amount of hydrogen peroxide. With an amount of 100 mM Bi_2_WO_6_ in the sol, an H_2_O_2_ concentration of 10.3 μM was observed. The mechanism of the film–water interface was postulated by Hoffmann et al. [102]. The particles on the surface absorb visible light, thus generating positively charged holes in the valence band (VB) and free electrons in the conduction band (CB), which is schematically described in Equation (1). Those combine with oxygen and generate ROS, which can recombine into H_2_O_2_ [99,102]. The processes involved are listed in Equations (2)–(5).

Bi_2_WO_6_ + hν → Bi_2_WO_6_ (e(CB)^−^ + h(VB)^+^) (1)

H_2_O ⇄ OH^−^ + H^+^ (2)

OH^−^ + h(VB)^+^ → OH^•^ (3)

O_2_ + e(CB)^−^ → O_2_^•−^ (4)

O_2_^•−^ + H^+^ → HO_2_^•^ (5)

2 OH^•^ → H_2_O_2_ (6)

**Figure 13:**
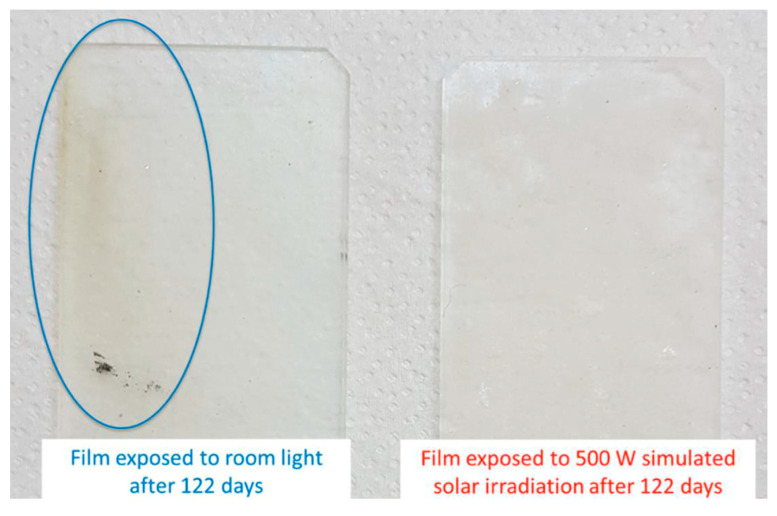
Irradiated films with a Bi_2_WO_6_ concentration of 50 mM showed no deposition of biomass after 122 days of irradiation [103].

As a compromise between efficacy and economics, a coating with 50 mM Bi_2_WO_6_ was tested in seawater with no additional irradiation and with simulated solar light [103]. After 122 days of irradiation, the residual water was analyzed. In comparison with the nonirradiated samples, the irradiated water samples showed an increase in the TOC (total organic carbon) and a decrease in the IC (inorganic carbon)-value. This can be explained by the growth of phytoplankton, which uses photosynthesis to consume inorganic carbon. It is noticeable that the room-light-irradiated samples showed a deposition of biomass on the layer (see Figure 13) as a result of the antifouling properties under solar irradiation.

**Figure 14:**
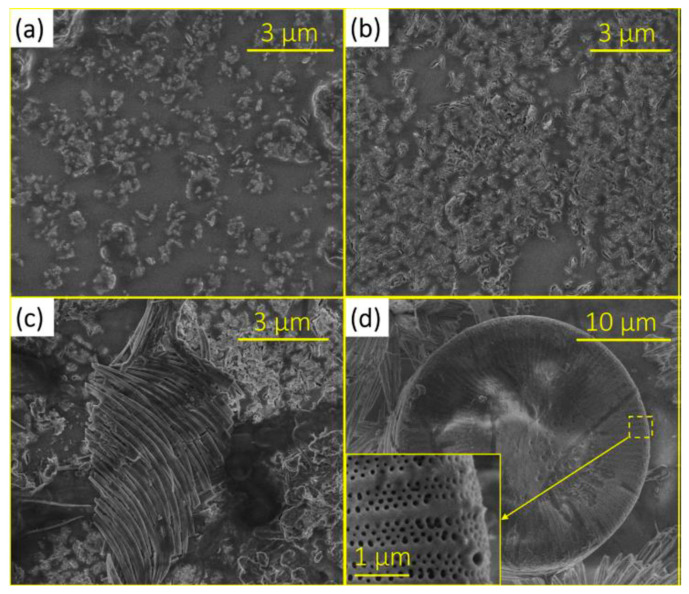
The SEM images of the different surfaces show big differences before and after immersion tests for 122 days. In (**a**), the image of the coated glass before the experiment can be seen. The additional irradiated sample, which can be seen in (**b**), just shows little differences from (**a**). The sample with no additional irradiation (**c**) shows a lot more growth on the surface, while the uncoated glass (**d**) shows the formation of large biological objects such as diatoms [103].

The decrease in microbiological growth on the coated glass after irradiation with simulated solar light can also be verified by the comparison of SEM images of the surfaces (Figure 14). These results, under laboratory conditions, show that the incorporation of Bi_2_WO_6_ in sol–gel-based layers consisting of MTES and TEOS improves the antifouling capabilities of the coating due to the formation of hydrogen peroxide under irradiation with simulated solar light.

To improve the practical applicability of the coating, the MTES was substituted by 1% C18-TMS and 49% C8-TES. In addition to that, 2-propanol was used as a solvent for the hydrolysis of the silanes. The resulting coatings showed large improvements in adhesion and hydrophobicity [104].

The formation of ROS can also be achieved by using zinc oxide. V. Panaite et al. used zinc oxide nanoparticles combined with GPTMS in an epoxy resin matrix applied to steel substrates [105]. The sol was prepared by sonication of commercial ZnO nanoparticles in a solution of GPTMS in tetrahydrofuran as solvent. The sol produced was then added to the liquid epoxy resin formulation in a proportion of one to ten percent. The resulting formulation was applied to the substrates by dip-coating and initially cured for 30 min at room temperature, followed by 30 min at 90 °C [105].

**Figure 15:**
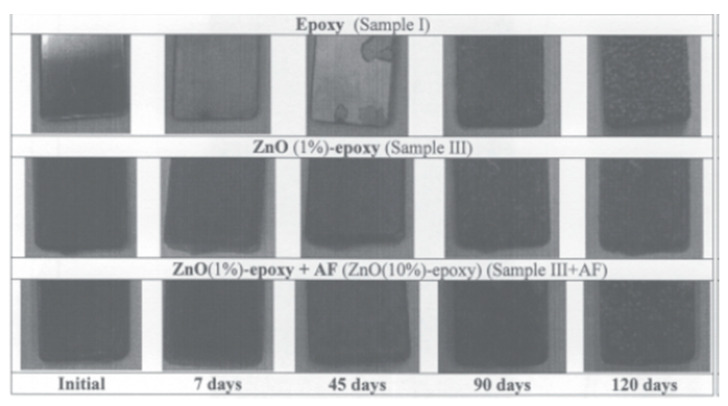
The addition of ZnO nanoparticles improved the resistance of the used substrates against marine fouling according to the visible state of the layers [105].

In order to test the antifouling properties of the coating, the coated panels were placed close to the surface in the Black Sea for a total of 120 days. For comparison, panels with the epoxy resin without the addition of ZnO nanoparticles were also tested. The influence of the ZnO-retaining formulation could be observed by the reduced growth of organisms when viewed with the naked eye. The influence of zinc oxide can be seen visually in Figure 15.

**Figure 16:**
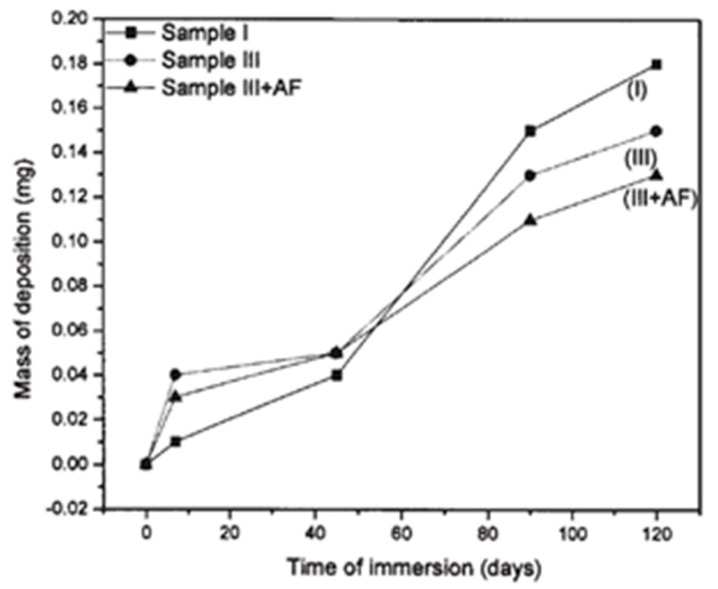
The antifouling properties of the ZnO nanoparticles can be seen from the decrease in the deposited biomass on the coatings [105].

The mass of the biological deposits was reduced from around 0.18 mg for the ZnO-free substrates to around 0.12 mg when ten percent ZnO was used. This effect is shown graphically in Figure 16.

**Figure 17:**
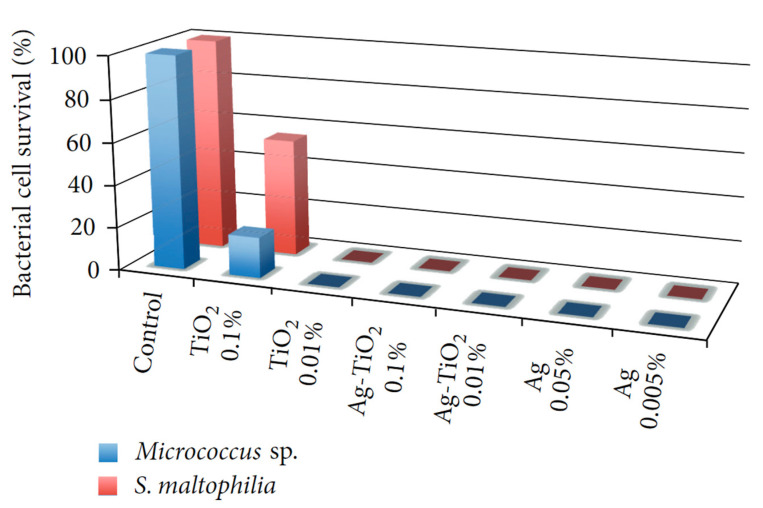
Incorporation of only 0.01% of the TiO_2_–nanoparticles synthesized by sol–gel synthesis led to complete killing of the tested bacteria [106].

Another antifouling agent that relies on photocatalytic activity is titanium dioxide, which can be easily prepared by sol–gel synthesis. Ruffolo et al. used a TiO_2_- sol that was prepared by hydrolysis of tetrabutyl orthotitanate (TBOT) in ethanol with nitric acid. After drying, the TiO_2_ nanoparticles were finished by calcination. Silver-doped TiO_2_ nanoparticles were synthesized similarly by adding 5 wt% silver nitrate to the hydrolysis solution [106]. Powder XRD measurements showed the formation of both TiO_2_ modifications, rutile and anatase, in the nanoparticle. In particular, anatase is known for its photocatalytic activity, which makes it useful for antifouling applications based on photocatalytic fragmentation of water [107]. The antibacterial properties of the doped and undoped nanoparticles were tested under laboratory conditions by exposing a solution of 1∙10^6^ cells/mL solution of *Stenotrophomonas maltophilia* and *Micrococcus* to 20 h of solar lamp irradiation with varying amounts of nanoparticles suspended in the solution. After incubation, the survival percentage was calculated as colony-forming units per ml. The results of this experiment are visualized in Figure 17.

To produce a coating, the nanoparticles were dispersed in a solution of 5 wt% “Paraloid B72” (EMA—methylacrylate copolymer) in acetone with polymer/nanoparticle ratios of 1:1, 1:10, and 1:100. The resulting dispersion was used for the coating of marble slabs. Marble was chosen due to the relevance of marine fouling for archaeological sites underwater. The coated slabs were tested in an immersion test with marine water under natural irradiation with sunlight and compared with uncoated slabs. The results show a significant decrease in microbiological colonization on the coated slabs with 1/10 nanoparticle/polymer ratio compared with uncoated slabs. This shows the potential of TiO_2_ as an antifouling agent with photocatalytic activity.

A coating consisting of TiO_2_ nanoparticles from *Evonik* incorporated in TEOS/C8-TES xerogel matrix was tested in a four-year immersion test on the mortar seatings of a Roman theatre. The treated quadrants showed significantly less fouling compared with the untreated quadrants, resulting in 61% less biological fouling [108].

A different approach to catalytic active antifouling coatings was taken and patented by Detty et al. by using dendrimeric organochalcogeno derivatives integrated into an organosilicon sol–gel matrix [109,110]. Since the natural concentration of hydrogen peroxide in surface-near seawater of about 1–2∙10^−7^ mol/L is not enough to observe antifouling properties, its properties as an oxidizing agent can be used to convert common species into near-surface biocides.

Selenium and tellurium derivatives of dendrimers are known for their catalytic activity of the bromide oxidation by hydrogen peroxide [111]. Therefore, they mimic the function of bromo peroxidases. The main product of this oxidation is hypobromous acid, which acts as a biocide. The oxidation of sodium bromide is described in Equation (7).

H_2_O_2_ + NaBr → HOBr + NaOH (7)

**Figure 18:**
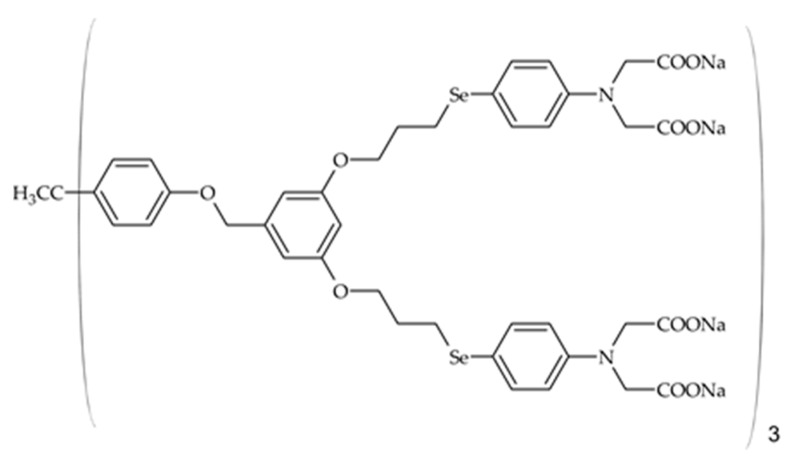
Example of a selenium-containing dendrimer used for antifouling coatings by Detty et al. [104].

Because of the average bromide concentration in seawater of about 1 mmol/L, this oxidation can take place reliably in low depths. The low natural concentration of hydrogen peroxide in seawater ensures that the concentration of hypobromic acid does not get critical for larger marine species [22]. One example of the used dendromeric organochalcogenes is given in Figure 18.

The sol–gel matrices used for the incorporation of the dendrimers consist of TEOS, *n*-Propyl-trimethoxysilane, TMOS, and bis(3-(Trimethoxysilyl)propyl)ethylenediamine. The catalysts were incorporated at amounts between 10 and 100 ppm. The covalent linking of hydroxy-functionalized dendrimer catalysts with silanols formed during the sol–gel process of the matrix is reported. In particular, telluride dendrimers showed a significant influence on grown algae spores on the sample surface in field tests [110]. A list of catalytic active components used for sol–gel coatings with antifouling properties is given in Table 3.

## 5. Conclusions

This review examines the advancement and utilization of sol–gel coatings as antifouling coatings, highlighting their ecological significance, particularly in marine environments where fouling significantly impacts ship surfaces. The authors of this review summarize the literature, primarily focusing on developments over the last ten years. There are different mechanisms for preventing the growth of fouling organisms. In the cited studies, the molecular design and nanoarchitectonics of inorganic–organic hybrid sol–gel systems are employed as key tools. One possibility is the integration of different siloxanes (for example, different lengths of alkyl chains (C3, C8, or C18)) and other molecules to develop surfaces that can either prevent fouling or facilitate the easy detachment of organisms that have stuck to them. Another point discussed in the review is the examination of surface topologies, which assess the impact of surface features and alterations on inhibiting organism adhesion. Moreover, the utilization of diverse additives, including silver, copper, and zinc nanoparticles, is explored to augment antifouling characteristics through their antimicrobial qualities. In addition, by incorporating active components such as ZnO or Bi_2_WO_6_ into the sol–gel coating, reactive products, primarily so-called ROS (reactive oxygen species), can be generated in situ. Several research reports are listed that showcase the effectiveness of various sol–gel formulations in minimizing fouling and inhibiting microbial proliferation. The findings of the studies are based on contact angle measurements, antimicrobial testing, and extended marine exposure experiments. Overall, the use of sol–gel coatings has many benefits, such as their robust chemical stability, versatile adaptability, simple synthesis at room temperature, and cost-effectiveness. The review offers a comprehensive analysis of existing antifouling technologies, with a specific emphasis on the potential and efficacy of sol–gel coatings in reducing the negative effects of marine fouling on the environment and economy.

## Figures and Tables

**Table 1:** Overview of additives incorporated into sol–gel matrices to achieve antifouling and fouling release effects.

Additive		Effect	Matrix	Reference
Nanoparticles	Cu, Zn, Ag	antibacterial activities	MTES	[3]
	ZnO	antimicrofouling	TEOS	[6]
	Ag + GO Ag	antibacterial activities antibacterial activities	TEOS/MTMO/PDMS SiO_2_/HPMC/Ag	[4,7]
Metaloxides	Cu_2_O	combat biofouling	MTMS/BTMS	[5]
Polysaccharides	Chitosan	antimicrobial activity	TEOS/GPTMS	[8]
		antifouling and fouling release	TEOS	[9]
	Alginate	antifouling and fouling release		
	Hyaluronic acid	antifouling and fouling release		
	Heparin Chitosan Alginic acid Hyaluronic acid Chondroitin sulfate	antimicrobial activity antifouling and fouling release antifouling and fouling release antifouling and fouling release antifouling and fouling release	MTES/ISTES	[9,10]
Enzymes	*Subtilisin*	antifouling	Glycerol/GPTES/PTEO/MPDMO/MTEO/N-Methylaminopropyltrimethoxysilan	[11]
	*B. licheniformis* + ZAPP + MOLY	anticorrosion and antifouling	TEOS/GPTMS/PDMS-amino	[12]
	*Paenibacillus polymyxa* + Molywhite^®^ 101-ED and Heucophos Zapp^®^	anticorrosion and antifouling	TEOS/MTES	[13]
Others	Eugenol	Biofilm inhibition and antifouling	TEOS	[14]

**Table 3:** Comparison of several additives for sol gel coatings with antifouling properties due to active catalysis.

Catalytic Active Component	Catalyzed Reaction	Antifouling Mechanism	Ref.
Bi_2_WO_6_ nanoparticles	Photolysis of water	Oxidation of biofilms by hydrogen-peroxide and ROS	[99,103,104]
ZnO nanorods	Photolysis of water	Oxidation of biofilms by hydrogen-peroxide and ROS	[105]
TiO_2_ nanoparticles	Photolysis of water and oxygen	Oxidation of biofilms by ROS	[106,108]
dendrimeric organochalcogeno-derivatives	Oxidation of halide ions to hypohalous acids	Biocidal properties of hypohalous acids	[22,109,110]

## Abbreviations

**Chemicals**	
APTES	3-Aminopropyltriethoxysilane
APTMS	(3-Aminopropyl)trimethoxysilane
BITS	2-(2-Hydroxy-3-(3-(trimethoxysilyl)propoxy)propyl)benzo[d]isothiazol-3(2H)-on
BMA	Butyl methacrylate
BSA	*bovine serum albumin*
BTMS	Butyltrimethoxysilane
C3-TMS	*n*-Propyltrimethoxysilane
C8-TES	*n*-Octyltriethoxysilane
C18-TMS	*n*-Octadecyltrimethoxysilane
COE	Carboxyethylsilanetriol
DBTDL	Dibutyltin dilaurate
DFMA	Dodecafluoroheptyl methacrylate
DMA	Dodecyl methacrylate
DMAP	3-Dimethylaminopropyltrimethoxysilane
EMA	Ethyl methacrylate
F16	Glycidyl-2,2,3,3,4,4,5,5,6,6,7,7,8,8,9,9-hexadecafluorononylether
FMA	1H,1H,2H,2H-Heptadecafluorodecyl methacrylate
FPES	Fluoropolyether silane (Fluorolink S10, Solvay Solexis)
GO	Graphene oxide
GPTMS	(3-Glycidyloxypropyl)trimethoxysilane
GPTES	(3-Glycidyloxypropyl)triethoxysilane
HFBM	2,2,3,4,4,4-hexafluorobutyl methacrylate
HMA	Hexyl methacrylate
HMPF	2-Hydroxy-2-Methylpropiophenone
HPMC	SiO_2_-hydroxypropylmethyl
ISTES	3-(1-(3-(Triethoxysilyl)propyl)-4,5-dihydro-1H-imidazol-3-ium-3-yl)propane-1-sulfonate
KH580	3-mercaptopropyl triethoxysilane
LBLHPS	Layer-by-layer assembling of hybrid polymer coatings
MAP	(3-Methylaminopropyl)trimethoxysilane
MAPS	(N-Methoxyacylethyl)-3-aminopropyltriethoxysilane
MAPTMS	3-(Trimethoxysilyl)propyl methacrylate
MMA	Methyl methacrylate
MOLY	Molywhite^®^ 101-ED
MPDMO	Methylphenyldimethoxysilane
MTAcS	Methyltrieacetoxysilane
MTES	Methyltriethoxysilane
MTEO	3-Mercaptopropyltriethoxysilane
MTMO	3-Mercaptopropyltrimethoxysilane
MTMS	Methyltrimethoxysilan
OMA	Octyl methacrylate
ORMOSIL	organically modified silicate
PDMS	Poly(dimethylsiloxane)
PDMSE	Poly(dimethylsiloxane) elastomer
PEG	Polyethylene glycol
PEGMA	Poly(ethyleneglycol)methylether- methacrylate
PEG-TMS	Poly(ethylene glycol) siloxane
PEOTMS	3-(Methoxy(polyethylenoxy))-propyltrimethoxysilane
PFOTES	1H,1H,2H,2H-Perfluorooctyltriethoxysilane
PH	Phenyltriethoxysilane
ROS	Reactive oxygen species
SBMA	[(methacryloyloxy) ethyl] dimethyl-(3-sulfopropyl) ammonium hydroxide
SBSi	Sulfobetaine silane
TBOT	Tetrabutyl orthotitanate
TBAF	Tetrabutylammonium fluoride
TDF	(Tridecyfluoro-1,1,2,2-tetrahydrooctyl)-triethoxysilane
TEOS	Tetraethoxysilane
TFP	(3,3,3-Trifluoropropyl)trimethoxysilane
TMOS	Tetramethoxysilane
TMAP	3-(Trimethoxysilyl)propyl- trimethylammonium-iodide
TPOZ	Tetrapropyl zirconate
TTIP	Titanium tetraisopropoxide
VTMS	Vinyltrimethoxysilane
ZAPP	Heucophos Zapp^®^
**Methods and Micellaneous**	
ASW	Artificial seawater
CA	Contact angle
HCA	Hexadecane contact angle
HSA	Hexadecane sliding angle
SFE/γ_S_	Surface energy
SFT/γ_T_	Surface tensions
UOWCA	Underoil water contact angle
UWOCA	Underwater oil contact angle
WCA	Water contact angle
WSA	Water sliding angle
CB	Conduction-band
EIS	Electrochemical impedance spectroscopy
IC	Inorganic carbon
ICP-AES	Inductively coupled plasma atomic emission spectroscopy
NMR	Nuclear magnetic resonance
SEM	Scanning electron microscopy
SPR	Surface plasmon resonance measurements
TGA	Thermogravimetric analysis
TOC	Total organic carbon
VB	Valence-band
XPS	X-ray photoelectron spectroscopy
µ	Relative friction coefficient
